# Aromatic secondary amine-functionalized fluorescent NO probes: improved detection sensitivity for NO and potential applications in cancer immunotherapy studies†
†Dedicated to Prof. Jin-Pei Cheng on the occasion of his 70th birthday.
‡
‡Electronic supplementary information (ESI) available: Synthesis, experimental procedures, supplemental spectra and imaging data, and ^1^H-, ^13^C-NMR, and MS spectra. See DOI: 10.1039/c8sc03694b


**DOI:** 10.1039/c8sc03694b

**Published:** 2018-10-03

**Authors:** Yingying Huo, Junfeng Miao, Junru Fang, Hu Shi, Juanjuan Wang, Wei Guo

**Affiliations:** a School of Chemistry and Chemical Engineering , Shanxi University , Taiyuan 030006 , China . Email: guow@sxu.edu.cn; b Scientific Instrument Center , Shanxi University , Taiyuan 030006 , China

## Abstract

Tumor-associated macrophages (TAMs), constituting up to 50% of the solid tumor mass and commonly having a pro-tumoral M2 phenotype, are closely associated with decreased survival in patients. Based on the highly dynamic properties of macrophages, in recent years the repolarization of TAMs from pro-tumoral M2 phenotype to anti-tumoral M1 phenotype by various strategies has emerged as a promising cancer immunotherapy approach for improving cancer therapy. Herein, we present an aromatic secondary amine-functionalized Bodipy dye **1** and its mitochondria-targetable derivative **Mito1** as fluorescent NO probes for discriminating M1 macrophages from M2 macrophages in terms of their difference in inducible NO synthase (iNOS) levels. The two probes possess the unique ability to simultaneously respond to two secondary oxides of NO, *i.e.*, N_2_O_3_ and ONOO^–^, thus being more sensitive and reliable for reflecting intracellular NO than most of the existing fluorescent NO probes that usually respond to N_2_O_3_ only. With **1** as a representative, the discrimination between M1 and M2 macrophages, evaluation of the repolarization of TAMs from pro-tumoral M2 phenotype to anti-tumoral M1 phenotype, and visualization of NO communication during the immune-mediated phagocytosis of cancer cells by M1 macrophages have been realized. These results indicate that our probes should hold great potential for imaging applications in cancer immunotherapy studies and relevant anti-cancer drug screening.

## Abstract 2

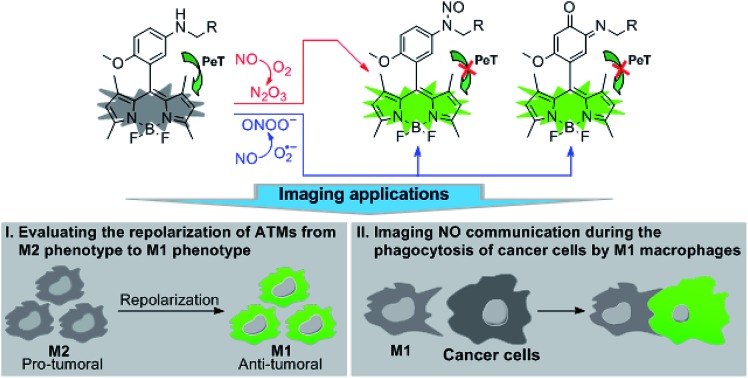
Fluorescent NO probes reported herein display high sensitivity for NO by responding to both N_2_O_3_ and ONOO^–^ and robust abilities for evaluating the repolarization of tumor-associated macrophages (TAMs).

## Introduction

Macrophages are specialized immune cells found all over the body that exist primarily to engulf and digest cellular debris, foreign substances, microbes, and cancer cells in a process called phagocytosis. Macrophages are particularly active in inflammation and infection, under which conditions, blood monocytes are recruited into the tissue where they differentiate into macrophages.^1^ Notably, macrophages are the most well-characterized type of tumor-infiltrating immune cells, and play crucial roles from anti-tumor to tumor progression and metastasis. Accumulating evidence reveals that the dual functions of macrophages can be attributed to their ability to adapt to the macroenvironment that leads to two main polarized phenotypes, *i.e.*, classically activated M1 macrophages and alternatively activated M2 macrophages.^2^–^4^ M1 macrophages, characterized by the expression of high-level inducible nitric oxide (NO) synthase (iNOS) as well as some pro-inflammatory cytokines, such as IL-12,^5^–^7^ have high bactericidal and tumoricidal activity partially due to their ability to secrete high levels of reactive oxygen/nitrogen species (ROS/RNS), such as hydrogen peroxide (H_2_O_2_), superoxide (O_2_^–^˙), and NO and its secondary metabolites dinitrogen trioxide (N_2_O_3_) and peroxynitrite (ONOO^–^). In contrast, M2 macrophages, characterized by the expression of arginase 1 (Arg-1) and anti-inflammatory cytokines, such as IL-10, commonly have a low level of iNOS^5^–^7^ and can assist tumor development by inducing angiogenesis, remodeling the extracellular matrix, stimulating cancer cell proliferation, and inhibiting adaptive immunity. In fact, under cancer-initiating conditions, the infiltrated macrophages have an M1 phenotype and are anti-tumoral; however, their continued presence in a tumor microenvironment polarizes them to tumor-associated macrophages (TAMs), which commonly have an M2 phenotype and are closely associated with decreased survival in patients due to their pro-tumoral role.^2^–^4^ Of note, the polarization of macrophages is a highly dynamic process and the phenotype of M1- or M2-polarized macrophages can be reversed depending on the microenvironmental cues they receive.^8^ For instance, the reversion of macrophages from M2 phenotype to M1 phenotype and reduction of immunosuppressive effects from the M2 population have been observed when TAMs were treated with interferon-γ/lipopolysaccharide (IFN-γ/LPS);^9^,^10^ in patients with extended survival, the M1 macrophages account for the majority of macrophages present within tumors,^11^ distinct from the cases in tumor development and metastasis, where macrophages predominantly exhibit a pro-tumoral M2 phenotype.^2^–^7^ Based on these discoveries, the repolarization of TAMs from M2 phenotype to M1 phenotype to activate their anti-tumoral potential by various strategies has emerged as an attractive and promising approach in cancer immunotherapy in recent years.^2^,^12^–^17^ In this context, the development of efficient methods that can discriminate M1 macrophages from M2 macrophages is of crucial guiding significance for cancer immunotherapy studies and relevant anti-cancer drug screening.

Although immunohistological quantification, enzyme linked immunosorbent assay (ELISA), and Western blot analysis of various biomarkers have routinely been used to distinguish between M1 and M2 macrophages,^5^–^7^,^17^ these methods are complex, time-consuming, and especially incompatible with living systems. By comparison, fluorescent probe-based techniques, which have become the gold standard for detection and imaging of various biological species in living systems, are the most promising to overcome these limitations due to their simplicity, convenience, sensitivity, noninvasiveness, and real-time spatial imaging capacity.^18^,^19^ However, the attractive techniques have never been exploited to identify M1 or M2 macrophages to date. Given that M1 macrophages express higher levels of iNOS than M2 macrophages, we envisioned that fluorescent NO probes when properly designed should have the potential to distinguish between M1 and M2 macrophages in terms of their difference in the iNOS level and thus the NO level.

Among various fluorescent NO probes,^20^–^28^ *o*-diamine-based ones, pioneered by Nagano's group, are by far the most often studied and applied fluorescent NO probes.^23^–^28^ The corresponding sensing mechanism is based on the reaction of the *o*-diamine group with the autoxidation product of NO, *i.e.*, dinitrogen trioxide (N_2_O_3_),^29^ to form the benzotriazole derivative, thereby triggering a fluorescence off–on response by inhibiting the photoinduced electron transfer (PeT) process. Although fluorescent NO probes of this kind have widely been applied in biological systems, some limitations still remain, such as possible interference by dehydroascorbic acid (DHA)/ascorbic acid (AA)/methylglyoxal (MGO)^30^–^33^ and a relatively long response time (commonly more than 5 min). To overcome these limitations, in recent years some new strategies have been actively developed, such as diazo ring formation,^34^,^35^ reductive deamination,^36^,^37^ monoprotection of vicinal diamine groups,^38^–^41^ aromatization of Hantzsch ester,^42^,^43^ and N-nitrosation of aromatic secondary amines.^44^–^47^ Yet despite the remarkable progress that has already been achieved, a widely overlooked issue is that almost all of these probes, including *o*-diamine-based ones, can reflect intracellular NO only by reacting with its autooxidation product N_2_O_3_, which would inevitably decrease the detection sensitivity for NO given that NO could also rapidly react with O_2_^–^˙ to generate ONOO^–^ at near diffusion control (∼10^10^ M^–1^S^–1^),^48^ and that these probes usually fail to give a fluorescence response toward ONOO^–^. The situation may be especially serious during the immune response of macrophages, where large amounts of NO and O_2_^–^˙ were simultaneously produced and coexisted with O_2_.^49^ Thus, the development of new fluorescent NO probes that can sensitively sense both N_2_O_3_ and ONOO^–^ is highly desired for improving not only the detection sensitivity of NO but also the reliability in distinguishing between M1 and M2 macrophages.

**Scheme 1:**
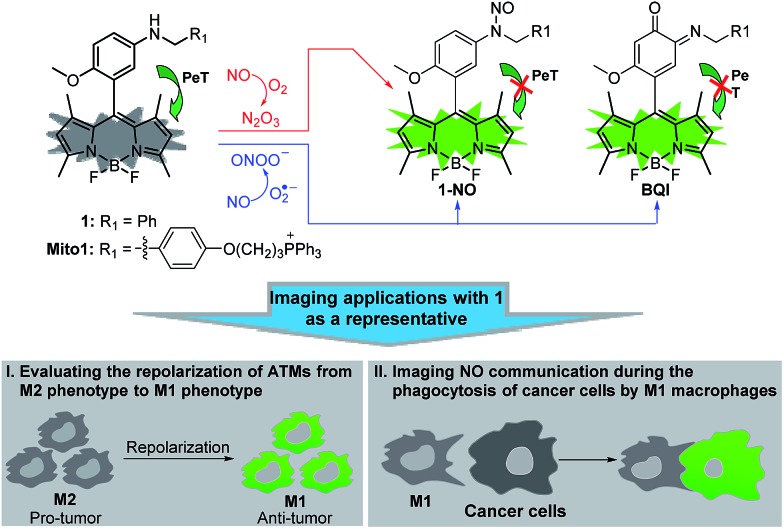
Proposed fluorescence sensing mechanisms of **1**/**Mito1** for N_2_O_3_ and ONOO^–^ and potential imaging applications of 1 as a representative in evaluating the repolarization of TAMs from M2 phenotype to M1 phenotype and imaging NO communication during the phagocytosis of cancer cells by M1 macrophages.

Recently, we reported for the first time that aromatic secondary amines could function as both the reaction group and PeT donor to construct fluorescent NO probes.^44^ However, like most of the previous reports, the as-obtained probe, *i.e.*, *N*-benzyl-4-hydroxyaniline-functionalized Bodipy, only exhibited a selective fluorescence off–on response toward N_2_O_3_ but not ONOO^–^. Further studies revealed that although not giving a fluorescence response, the probe could react with ONOO^–^ to lead to a nonfluorescent debenzylation product. This means that in practical bioimaging assays, the probe would probably suffer from the risk of being consumed by coexisting ONOO^–^, thereby resulting in decreased sensitivity for NO. However, to our delight, when *N*-benzyl-4-methoxyaniline was employed as the reaction group instead of the *N*-benzyl-4-hydroxyaniline group mentioned above, the newly developed fluorescent probe, *i.e.*, *N*-benzyl-4-methoxyaniline-functionalized Bodipy **1** and its mitochondria-targetable derivative **Mito1** (Scheme 1), not only overcame the shortcomings of classic *o*-diamine-type probes, such as possible interference by DHA/AA/MGO and a long response time, but also displayed a significant fluorescence off–on response for both N_2_O_3_ and ONOO^–^. The unique sensing properties endow the probes with high sensitivity for reflecting intracellular NO as indicated by their ability to image basal and endogenous NO in living cells. With **1** as a representative, we have successfully realized the discrimination between M1 and M2 macrophages and visualization of the repolarization of TAMs from M2 phenotype to M1 phenotype induced by IFN-γ/LPS. Also, we confirmed that during the immune-mediated phagocytosis of cancer cells by M1 macrophages, NO secreted by M1 macrophages could diffuse across the cancer cell membrane to exert its tumoricidal action by producing cytotoxic N_2_O_3_ and ONOO^–^. These findings strongly indicate that our probes should hold great potential for imaging applications in cancer immunotherapy studies and relevant drug screening.

## Results and discussion

### Synthesis and spectral response of **1** and **Mito1** for N_2_O_3_ and ONOO^–^

**Fig. 1:**
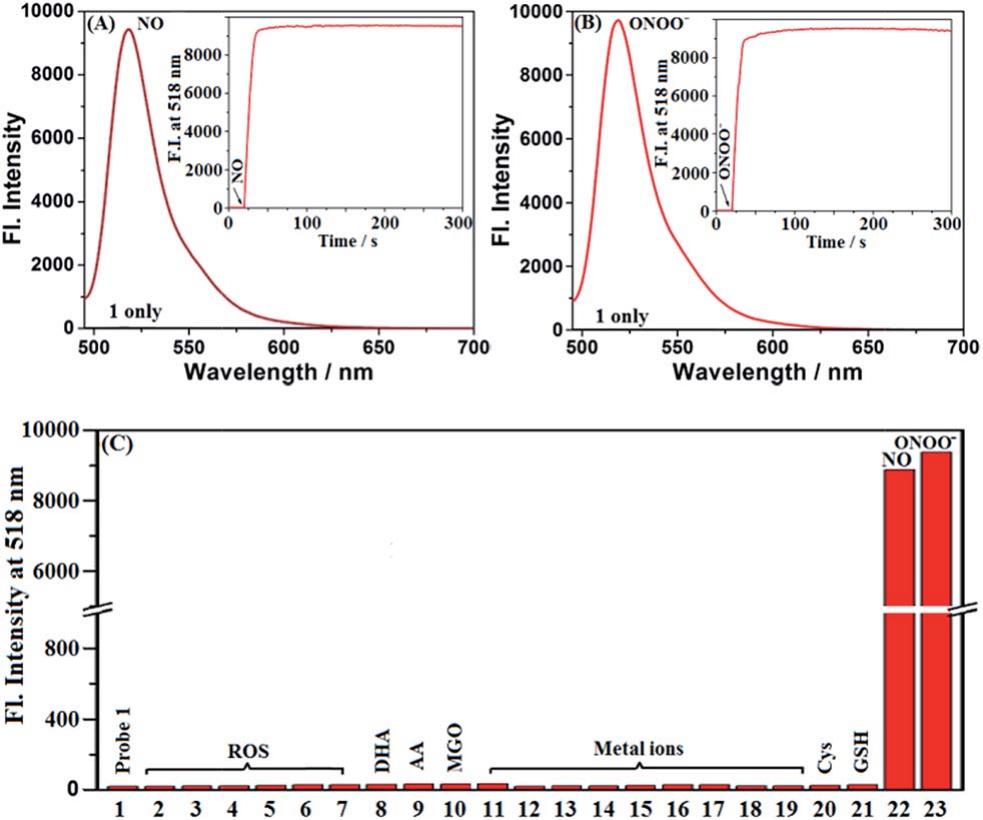
(A and B) Fluorescence spectra of 1 (4 μM) treated with and without NO (50 μM) or ONOO^–^ (20 μM) under aerobic conditions. Inset: the time-dependent fluorescence intensity changes of 1 (4 μM) treated with and without NO or ONOO^–^ (20 μM). (C) Fluorescence intensities of 1 (4 μM) treated with various competitive species at the time point of 2 min. (1) 1 only; (2) ClO^–^; (3) H_2_O_2_; (4) O_2_˙^–^; (5) ^1^O_2_; (6) HO˙; (7) NO_2_^–^; (8) DHA; (9) AA; (10) MGO; (11) K^+^; (12) Ca^2+^; (13) Na^+^; (14) Mg^2+^; (15) Al^3+^; (16) Zn^2+^; (17) Fe^2+^; (18) Fe^3+^; (19) Cu^2+^; (20) Cys; (21) GSH; (22) NO; (23) ONOO^–^. Concentrations for (2–7), 100 μM; for (8–21), 1 mM; for (22), 50 μM; for (23), 20 μM. Conditions: PBS (50 mM, pH 7.4, containing 20% CH_3_CN); *λ*_ex_ = 485 nm; *λ*_em_ = 518 nm; slits: 5/10 nm; voltage: 600 V.

Probes **1** and **Mito1** could be easily synthesized by a simple three-step procedure starting from commercially available 2-methoxy-5-nitrobenzaldehyde, including the initial synthesis of Bodipy dye, subsequent reduction of the nitro group to the amino group, and final reductive amination with corresponding benzaldehydes. The detailed synthesis and characterization data of **1** and **Mito1** are presented in the ESI.‡ With the two probes in hand, we first evaluated the spectral response of **1** for N_2_O_3_ and ONOO^–^ in PBS buffer (50 mM, pH 7.4, containing 20% CH_3_CN). As shown in Fig. 1A, the solution of **1** itself had an extremely poor fluorescence at 518 nm (*Φ* = 0.016), presumably due to PeT from the electron-rich *N*-benzyl-4-methoxyaniline unit to the excited Bodipy core; however, upon treatment with excess NO solution under aerobic conditions (conditions for producing N_2_O_3_),^23^–^28^ a significant fluorescence enhancement (880-fold) was observed from the dark background, indicating that the reaction of **1** with N_2_O_3_ could efficiently block the PeT process and thereby turn-on the fluorescence. Notably, as revealed by the kinetics study (Fig. 1A, inset), the fluorescence response of **1** for N_2_O_3_ was fairly fast and could be completed within 10 s, indicative of the potential of **1** for real-time imaging of endogenous N_2_O_3_ in biosystems. The fluorescence titration assay was further performed to evaluate the sensitivity of **1** for N_2_O_3_. As shown in Fig. S1 (ESI‡), a good linearity between the fluorescence intensities at 518 nm and the concentrations of added NO (0–16 μM) was observed, and the detection limit (DL) for N_2_O_3_ (4NO + O_2_ = 2N_2_O_3_) was calculated to be as low as 0.4 nM based on 3*σ*/*k*. When compared with the *N*-benzyl-4-hydroxyaniline-functionalized Bodipy probe reported by us previously,^44^**1** displays a bigger fluorescence off–on response and higher detection sensitivity for N_2_O_3_, indicating that the *N*-benzyl-4-methoxyaniline group of **1** should be a more excellent reaction group for N_2_O_3_ than the *N*-benzyl-4-hydroxyaniline group. Importantly, when **1** was treated with excess ONOO^–^ under the same conditions, a rapid and great fluorescence off–on response was also observed, which is almost consistent with the case of N_2_O_3_ in either fluorescence intensity or response kinetics (Fig. 1B). Moreover, a good linear correlation between the fluorescence intensities and the concentrations of ONOO^–^ in the range of 0–2.5 μM was also found (Fig. S2, ESI‡), and the DL for ONOO^–^ was calculated to be 0.14 nM based on 3*σ*/*k*. The results reveal that **1** is extremely sensitive not only for N_2_O_3_ but also for ONOO^–^, thus being very promising as a more sensitive indicator to reflect intracellular NO.

To establish the selectivity, we tested the fluorescence response of **1** toward various biologically relevant species, including reactive oxygen/nitrogen species (ROS/RNS: ClO^–^, H_2_O_2_, O_2_˙^–^, ^1^O_2_, ·OH, NO_2_^–^, NO, and ONOO^–^), DHA/AA/MGO, metal ions (K^+^, Ca^2+^, Na^+^, Mg^2+^, Al^3+^, Zn^2+^, Fe^2+^, Fe^3+^, and Cu^2+^), and biothiols (Cys and GSH). As shown in Fig. 1C, the treatment of **1** with either N_2_O_3_ (NO/O_2_) or ONOO^–^ could induce a significant fluorescence off–on response, while the other competitive species failed to give any obvious fluorescence alteration of **1**, indicating that the probe is highly specific for N_2_O_3_ and ONOO^–^. In addition, **1** was almost nonfluorescent in the pH range of 5–9, but displayed the best fluorescence response for N_2_O_3_ and ONOO^–^ at 7.4 (Fig. S3, ESI‡), thus being suitable for imaging application at physiological pH.

Encouraged by the above results, we further tested the fluorescence sensing performances of **Mito1** for both N_2_O_3_ and ONOO^–^ under the same conditions. Indeed, the probe was designed as a mitochondria-targetable fluorescent NO probe by installing a mitochondria-targeted triphenylphosphonium (TPP) cation^50^,^51^ to the molecular skeleton of **1**. Interestingly, as shown in Fig. S4–8 (ESI‡), **Mito1** displayed almost the same sensing performances for N_2_O_3_ and ONOO^–^ as **1**, such as the significant and rapid fluorescence off–on response, high selectivity and sensitivity, and excellent fluorescence response at physiological pH, indicating that the TPP cation rarely affects the sensing performances of **Mito1** toward N_2_O_3_ and ONOO^–^. Thus, **Mito1** should have the potential to sensitively and specifically reflect mitochondrial NO.

Overall, as revealed by the above assays, **1** and **Mito1** displayed high sensitivity, excellent selectivity, and fast response ability for both N_2_O_3_ and ONOO^–^ under the simulated physiological conditions, thus holding great potential for probing NO-related physiology and pathology.

### Sensing mechanisms of **1** and **Mito1** for N_2_O_3_ and ONOO^–^

**Scheme 2:**
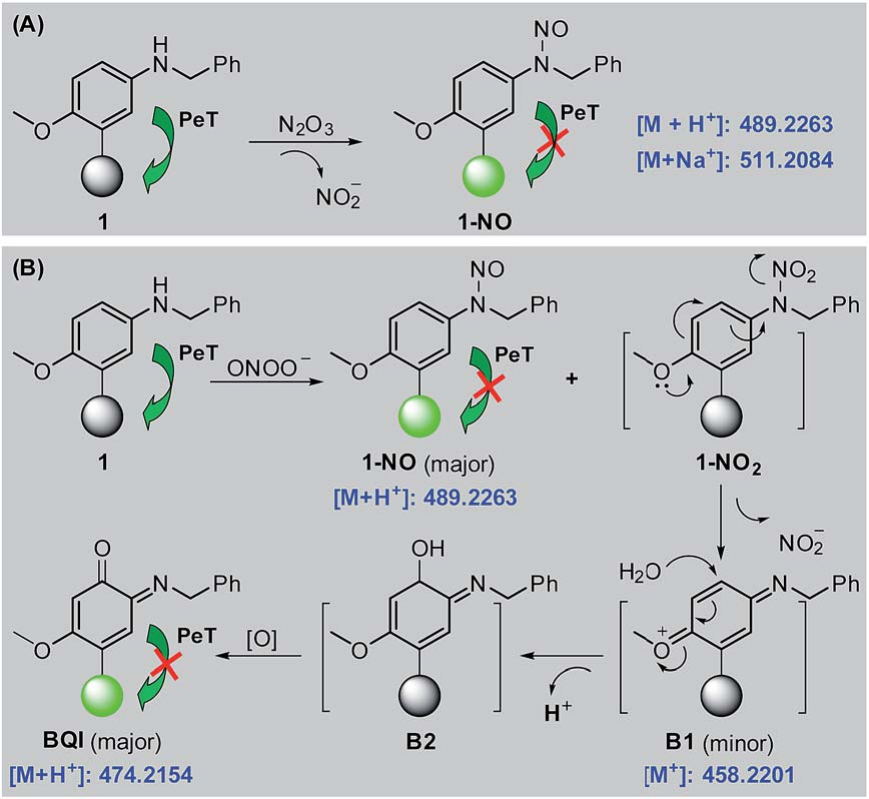
Proposed sensing mechanisms of 1 for N_2_O_3_ (A) and ONOO^–^ (B), respectively. Round balls represent the Bodipy core.

With **1** as a representative, we subsequently studied the sensing mechanisms of the probe for both N_2_O_3_ and ONOO^–^ by HPLC-HRMS assays. As shown in Fig. S9 (ESI‡), the HPLC analysis showed that the reaction of **1** with N_2_O_3_ mainly produced a new peak, which could be assigned to N-nitroso product **1-NO** in terms of HRMS data (*m*/*z* calcd for [M + H^+^] 489.2273, found 489.2263) (Scheme 2A). This is consistent with the previous report that aromatic secondary amines can react with NO under aerobic conditions to give the N-nitroso product.^44^ However, in the case of ONOO^–^, in addition to the major N*-*nitroso product **1-NO** (*m*/*z*: calcd for [M + H^+^] 489.2273, found 489.2263), two unknown new products, one major and the other minor, were observed as well in HPLC analysis (Fig. S10, ESI‡). Considering that secondary amines can react with ONOO^–^ to produce both N*-*nitroso and N*-*nitro products,^52^ we proposed a possible reaction mechanism as follows (Scheme 2B): first, the reaction of **1** with ONOO^–^ produced the *N-*nitroso product **1-NO** and N-nitro product **1-NO_2_**; due to the strong push–pull electronic interaction between the –OMe group and –NO_2_ group, **1-NO_2_** was unstable and underwent an intramolecular two-electron transfer to give *p*-benzoquinone imine intermediate **B1**; the intermediate was also unstable and could be attached by the H_2_O molecule *via* a Michael addition-like reaction to generate intermediate **B2**; the oxidative dehydrogenation of **B2** afforded *o*-benzoquinone imine **BQI** as the final product of the reaction pathway. According to the proposed mechanism, the above-mentioned two unknown products could reasonably be assigned to **B1** (minor) and **BQI** (major) in terms of the excellent matching of calculated and observed *m*/*z* values (for **B1**, calcd for [M^+^] 458.2215, found 458.2201; for **BQI**, calcd for [M + H^+^] 474.2164, found 474.2154) (Fig. S10, ESI‡). Thus, the HPLC-HRMS assays nicely support our proposed reaction mechanisms of **1** for N_2_O_3_ and ONOO^–^.

Further, the fluorescence off–on response of **1** for N_2_O_3_ and ONOO^–^ by inhibiting the PeT process was rationalized by the Frontier orbital energy diagrams of **1**, **1-NO**, and **BQI**, obtained by Becke's three-parameter hybrid exchange function with the Lee-Yang-Parr gradient-corrected correlation functional (B3LYP functional) and 6-31+G* basis set (Fig. S11, ESI‡). To support the conclusion, we studied the fluorescence changes of **1** in mixed water–glycerol systems (0–100% of glycerol) with varied viscosity. As shown in Fig. S12 (ESI‡), in these cases **1** still displayed negligible fluorescence, strongly indicating that no fluorescence of **1** is indeed due to the PeT process, rather than rotation or vibration-relevant nonradiative processes.^53^

### Basic imaging ability of **1** and **Mito1** for N_2_O_3_ and ONOO^–^ in living cells as well as their subcellular distribution

**Fig. 2:**
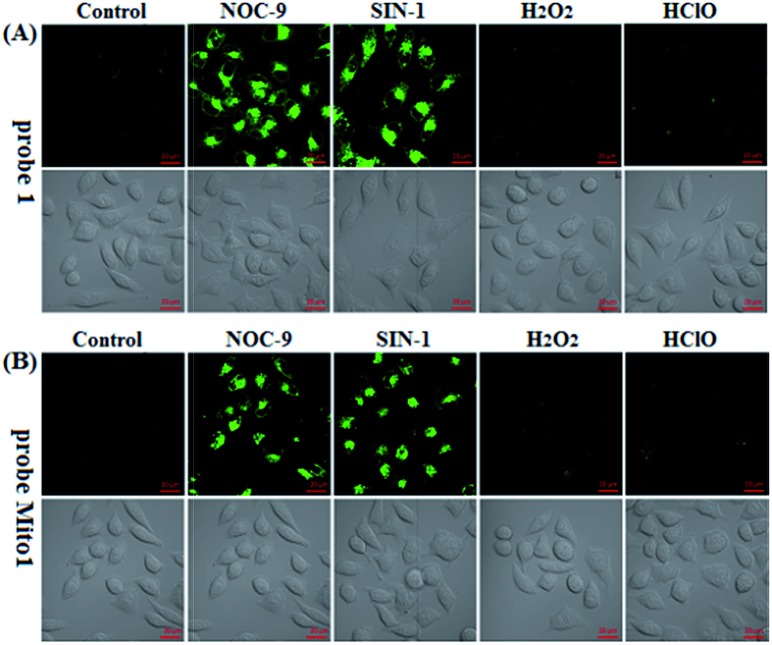
Confocal images of HeLa cells pretreated with **1** (2 μM) (A) or **Mito1** (2 μM) (B) for 20 min, and then treated with NOC-9 (25 μM), SIN-1 (10 μM), H_2_O_2_ (50 μM), and ClO^–^ (50 μM) for 20 min, respectively, in PBS. Emission was collected at 493–600 nm (*λ*_ex_ = 488 nm). Scale bar: 20 μm.

Prior to biological imaging applications, the cytotoxicity of **1** and **Mito1** was first tested in HeLa cells by MTT assays. As shown in Fig. S13 (ESI‡), after 24 h of cellular internalization of less than 8 μM of **1** or **Mito1**, >90% of the cells remained viable, indicative of the good biocompatibility of the two probes. Notably, **1** displayed an obviously lower cytotoxicity than **Mito1**, presumably due to its uncharged property reducing its interaction with either the negatively charged DNA or the mitochondrial membrane with highly negative potential. Even so, in order to reduce the interference to cell proliferation and physiology, a low concentration of **1** or **Mito1** (2 μM), survival rates close to 100% in the case, was used in the subsequent bioimaging assays. Subsequently, we evaluated the selectivity of **1** or **Mito1** for N_2_O_3_ and ONOO^–^ in human cervical cancer HeLa cells. As shown in Fig. 2, HeLa cells loaded with **1** or **Mito1** showed negligible background fluorescence; when the **1**- or **Mito1**-loaded HeLa cells were treated with NOC-9 (a commercial NO donor) or SIN-1 (a commercial ONOO^–^ donor), a strong intracellular green fluorescence was observed for both cases; when **1**- or **Mito1**-loaded HeLa cells were treated with representative ROS, such as H_2_O_2_ and ClO^–^, almost no any intracellular green fluorescence was found. The results suggest that **1** and **Mito1** still possess high specificity for N_2_O_3_ and ONOO^–^ in a cell environment.

**Fig. 3:**
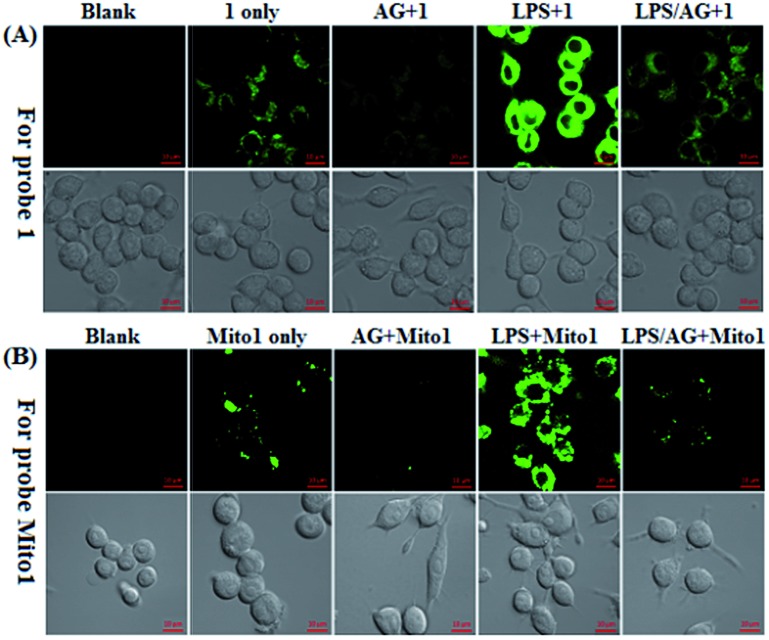
Confocal images of the basic and stimulator-induced NO in RAW 264.7 macrophages using **1** (A) and **Mito1** (B). For imaging of intracellular basal NO, cells were treated directly with **1** or **Mito1** (2 μM, 20 min) in PBS; for imaging of the stimulator-induced NO, cells were pretreated with stimulators LPS (20 μg mL^–1^)/INF-γ (150 units per mL) for 6 h in PBS and then treated with **1** or **Mito1** (2 μM, 20 min); for inhibition assays, cells were pretreated with LPS (20 μg mL^–1^)/INF-γ (150 units per mL) for 6 h in the presence of AG (0.5 mM) and then treated with **1** or **Mito1** (2 μM, 20 min). Emission was collected at 493–600 nm (*λ*_ex_ = 488 nm). Scale bar: 10 μm.

Encouraged by the above results, we further tested the ability of **1** or **Mito1** for imaging endogenous N_2_O_3_ and ONOO^–^ in mouse RAW264.7 macrophages that are known to express high-level iNOS upon stimulation by LPS/IFN-γ.^54^ As shown in Fig. 3, RAW264.7 cells themselves were nonfluorescent; upon incubation with **1** or **Mito1**, the cells displayed a weak yet clear intracellular green fluorescence; when the cells were pretreated with NO synthase inhibitor aminoguanidine (AG)^55^ and then treated with **1** or **Mito1**, the intracellular green fluorescence was greatly inhibited. The results indicate that the two probes are sensitive enough to determine the basal level of intracellular NO by responding to N_2_O_3_ or ONOO^–^. Further, when the cells were stimulated with LPS/IFN-γ and then treated with **1** or **Mito1**, a bright intracellular green fluorescence was clearly observed; when the cells were stimulated with LPS/IFN-γ in the presence of AG and then treated with **1** or **Mito1**, the intracellular green fluorescence was greatly inhibited. Thus, the two probes can be used to sense the LPS/IFN-γ-triggered outburst of endogenous NO, indicating their potential for studying various NO-related pathophysiological events.

**Fig. 4:**
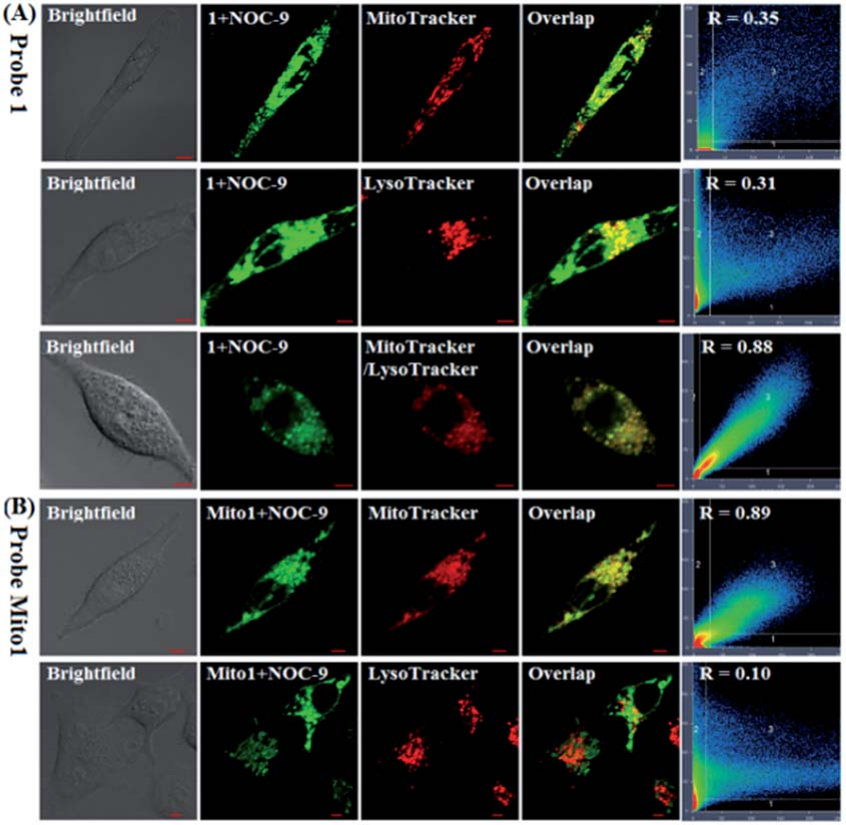
(A) Confocal images of HeLa cells co-stained with 1 (2 μM)/MitoTracker Red FM (0.3 μM), 1 (2 μM)/LysoTracker Deep Red (0.07 μM), and 1 (2 μM)/MitoTracker Red FM (0.3 μM)/LysoTracker Deep Red (0.07 μM) for 30 min, respectively, in PBS, and then treated with NOC-9 (30 μM, 20 min). (B) Confocal images of HeLa cells co-stained with **Mito1** (2 μM)/MitoTracker Red FM (0.3 μM) and **Mito1** (2 μM)/LysoTracker Deep Red (0.07 μM) for 30 min, respectively, in PBS, and then treated with NOC-9 (25 μM, 20 min). For **1** and **Mito1**, emission was collected at 493–600 nm (*λ*_ex_ = 488 nm); for MitoTracker and LysoTracker, emission was collected at 638–747 nm (*λ*_ex_ = 633 nm). Scale bar: 5 μm.

Also, we tested the subcellular distribution of **1** and **Mito1** in HeLa cells by costaining assays. In the assays, NOC-9 was used to light up the two probes in cells, and Pearson's correlation coefficient (*R*) was used to analyze the linear correlation of fluorescence signals between the green channel (for probes) and red channel (for commercial trackers). As shown in Fig. 4A, when HeLa cells were co-incubated with **1**/MitoTracker or **1**/LysoTracker followed by NOC-9 treatment, a poor overlapping image was observed for both cases (*R* = 0.35 and 0.31, respectively), indicating that **1** is not specific for either mitochondria or lysosomes. However, when HeLa cells were co-incubated with **1**, MitoTracker, and LysoTracker followed by NOC-9 treatment, we observed an excellent overlapping image from the green channel and red channel (*R* = 0.88), indicating that **1** was indeed distributed over both mitochondria and lysosomes. However, as shown in Fig. 4B, when HeLa cells were co-incubated with **Mito1**/MitoTracker or **Mito1**/LysoTracker followed by NOC-9 treatment, a good overlapping image along with a high Pearson's correlation coefficient was only observed for the former (*R* = 0.89) but not the latter (*R* = 0.10), indicating that **Mito1** could preferably localize in mitochondria rather than lysosomes. The excellent localization of **Mito1** in mitochondria could indeed be attributed to its lipophilic TPP cation that directs the probe into mitochondria by the highly negative potential of the mitochondrial membrane (about –180 mV).^50^,^51^

### Potential applications in cancer immunotherapy studies

**Fig. 5:**
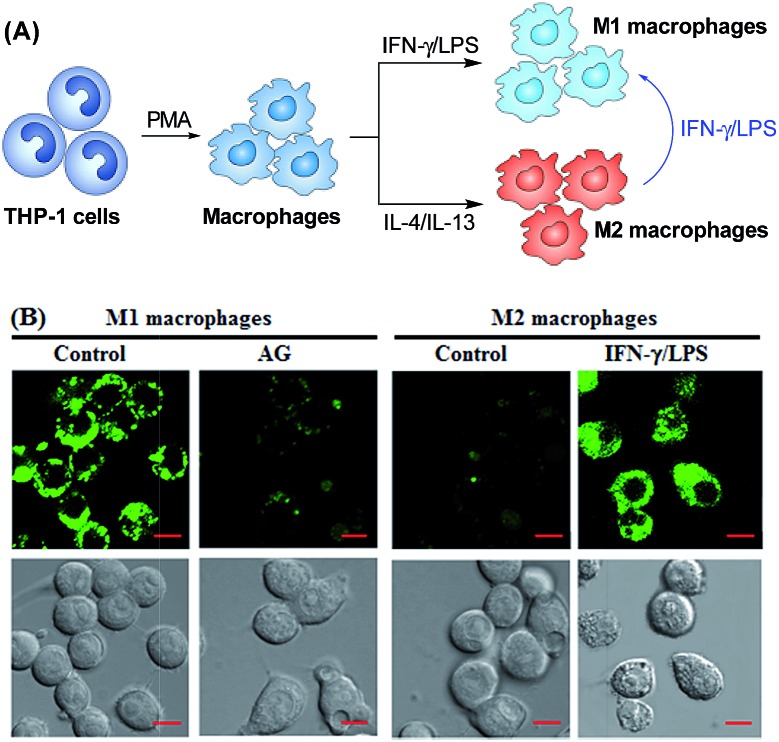
(A) Macrophages can be differentiated starting from the human monocytic cell line THP-1. Once differentiated in the presence of PMA, they can be polarized into M1 and M2 macrophages by IFN-γ/LPS and IL-4/IL-13 treatment, respectively. M1 macrophages could also be produced by the polarization of M2 macrophages by IFN-γ/LPS. (B) Confocal fluorescence images of M1 or M2 macrophages treated with 1 in the absence and presence of AG or IFN-γ/LPS. Emission was collected at 493–600 nm (*λ*_ex_ = 488 nm). Scale bar: 10 μm.

Having established their excellent imaging ability for NO in chemical systems and living cells by responding to both N_2_O_3_ and ONOO^–^, we envisioned that **1** and **Mito1** should have the potential to distinguish between M1 and M2 phenotypes in terms of their difference in the iNOS level and thus the NO level.^5^–^7^ To this end, we set up a model of human macrophage polarization according to a previously reported method.^3^ Briefly, human monocytic THP-1 cells were first differentiated into macrophages by 24 h incubation with phorbol 12-myristate 13-acetate (PMA) followed by 24 h incubation in RPMI medium; then, the macrophages were polarized in M1 macrophages by incubation with IFN-γ/LPS, and in M2 macrophages by incubation with interleukin 4 (IL-4) and interleukin 13 (IL-13) (Fig. 5A). After a thorough wash to remove all stimuli, the M1- and M2-polarized macrophages were treated with **1** (as a representative) and then imaged under a confocal laser scanning microscope. As shown in Fig. 5B, a bright intracellular green fluorescence could be observed in **1**-loaded M1 macrophages, but not in **1**-loaded M2 macrophages; moreover, when M1 macrophages were pretreated with NO synthase inhibitor AG and then treated with **1**, the intracellular green fluorescence was greatly inhibited. The results indicate that **1** could discriminate M1 macrophages from M2 macrophages in terms of their difference in the iNOS level and thus the NO level. Of note, when M2 macrophages were pretreated with IFN-γ/LPS and then treated with **1**, the intracellular green fluorescence was greatly recovered, consistent with the report that M2 macrophages could be repolarized to M1 macrophages by IFN-γ/LPS treatment.^9^,^10^

**Fig. 6:**
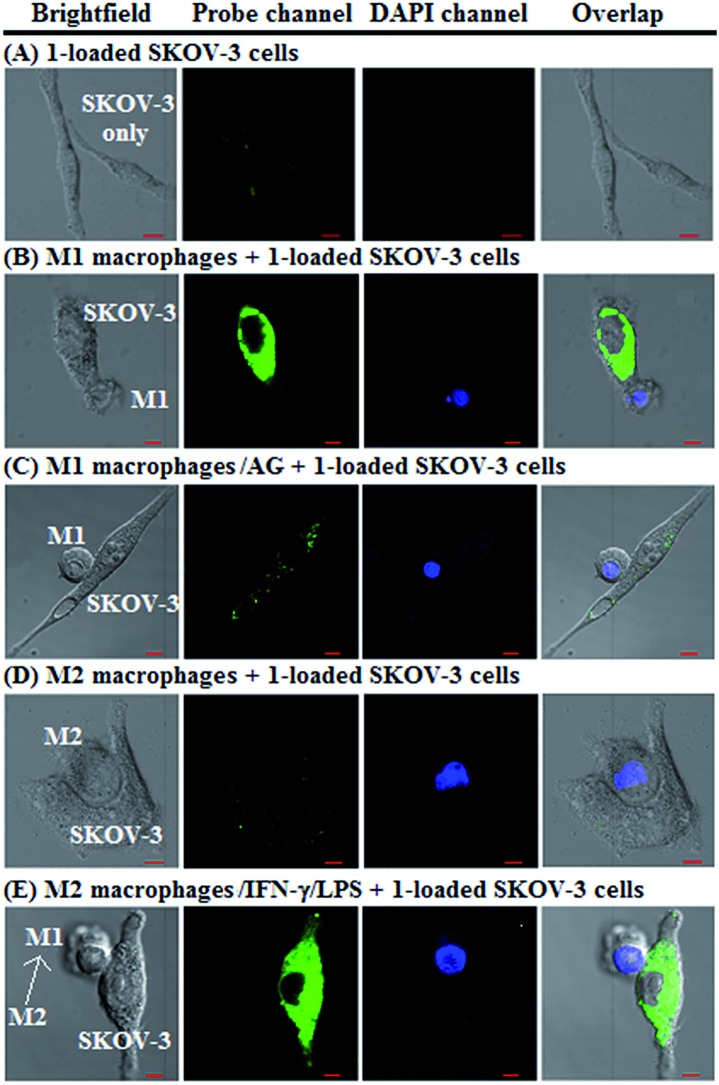
Imaging NO communication between DAPI-stained macrophages and 1-loaded SKOV-3 cancer cells in a co-culture system. (A) 1-loaded SKOV-3 cancer cells only (as a control); (B) 1-loaded SKOV-3 cells captured by DAPI-stained M1 macrophages in the co-culture system; (C) 1-loaded SKOV-3 cells captured by DAPI-stained and AG-pretreated M1 macrophages in the co-culture system; (D) 1-loaded SKOV-3 cells captured by DAPI-stained M2 macrophages in the co-culture system; (E) 1-loaded SKOV-3 cells captured by DAPI-stained and IFN-γ/LPS-pretreated M2 macrophages in the co-culture system. For the probe channel, emission was collected at 493–600 nm (*λ*_ex_ = 488 nm); for the DAPI channel, emission was collected at 410–485 nm (*λ*_ex_ = 405 nm). Scale bar: 5 μm.

Finally, we tested the ability of **1** to image NO communication during the phagocytosis of cancer cells by macrophages in a co-culture system containing M1- or M2-polarized macrophages and SKOV-3 human ovarian cancer cells. Briefly, the M1- or M2-polarized macrophages were first stained with commercial blue-fluorescent nucleus dye DAPI, and then co-cultured with **1**-loaded SKOV-3 cells for 12 h, followed by imaging under a confocal laser scanning microscope. Note that the use of DAPI dye is in order to distinguish M1 or M2 macrophages from SKOV-3 cells in the co-culture system. As shown in Fig. 6A, the SKOV-3 cells cultured alone displayed almost no intracellular fluorescence in the green channel when treated with **1**, indicating that the cancer cells express negligible intracellular NO. Upon capture by M1 macrophages in the co-culture system, the **1**-loaded SKOV-3 cells exhibited a strong intracellular green fluorescence (Fig. 6B), indicating that during the immune-mediated phagocytosis of cancer cells, NO secreted by M1 macrophages could diffuse across the cell membrane of cancer cells to exert its tumoricidal influence by producing cytotoxic N_2_O_3_ and ONOO^–^. The result was further supported by an inhibition assay, where the **1**-loaded SKOV-3 cells captured by the AG-pretreated M1 macrophages displayed an obviously decreased intracellular green fluorescence (Fig. 6C). In sharp contrast, when the **1**-loaded SKOV-3 cells were captured by M2 macrophages in the co-culture system, almost no intracellular green fluorescence could be observed in the former (Fig. 6D), in line with the low iNOS level in M2 macrophages. However, when M2 macrophages were pretreated with IFN-γ/LPS and then co-cultured with **1**-loaded SKOV-3 cells, the captured SKOV-3 cells displayed a dramatically increased intracellular green fluorescence (Fig. 6E), confirming that M2 macrophages can be polarized to M1 macrophages by IFN-γ/LPS.^9^,^10^

Overall, the above results confirm that **1** could be used to discriminate M1 macrophages from M2 macrophages, evaluate the repolarization of TAMs from pro-tumoral M2 phenotype to anti-tumoral M1 phenotype, and image NO communication between macrophages and cancer cells during the immune-mediated phagocytosis process, thus being very promising for imaging applications in cancer immunotherapy studies and relevant anti-cancer drug screening.

## Conclusions

In summary, we in this work presented an aromatic secondary amine-functionalized Bodipy dye **1** and its mitochondria-targetable derivative **Mito1** as highly sensitive fluorescent NO probes for discriminating M1 macrophages from M2 macrophages in terms of their difference in the iNOS level and thus the NO level. The high sensitivity of the two probes for NO originates from their unique ability to simultaneously respond to N_2_O_3_ and ONOO^–^. In this regard, the two probes should be superior to most of the existing fluorescent NO probes that can only respond to N_2_O_3_. Although an *o*-diamine-locked rhodamine lactam derivative has been reported to be able to give a similar fluorescence off–on response for both N_2_O_3_ and ONOO^–^,^38^ the later studies found that this type of probe could suffer from serious interference by intracellular abundant Cys to lead to decreased sensitivity for NO.^56^ Importantly, with **1** as a representative, we have successfully realized the discrimination of M1 macrophages from M2 macrophages, evaluation of the repolarization of TAMs from pro-tumor M2 phenotype to anti-tumor M1 phenotype, and visualization of NO communication during the immune-mediated phagocytosis of cancer cells by M1 macrophages. Thus, our probes should hold great potential for NO-related physiological and pathological studies as well as anticancer drug screening in cancer immunotherapy.

## Conflicts of interest

There are no conflicts to declare.

## Supplementary Material

Supplementary information
