# Supplementary material for: Aromatic secondary amine-functionalized fluorescent NO probes: improved detection sensitivity for NO and potential applications in cancer immunotherapy studies
Source: Chem Sci. 2018 Oct 3;10(1):145–52. doi: 10.1039/c8sc03694b (PMC6328002; doi:10.1039/c8sc03694b)
Supplement: Supplementary file 1 [file SC-010-C8SC03694B-s001.pdf]

Electronic Supplementary Material (ESI) for Chemical Science.
This journal is © The Royal Society of Chemistry 2018

Supporting Information

Aromatic secondary amine-functionalized fluorescent NO probes: improved detection sensitivity for NO and potential applications in cancer immunotherapy studies

Yingying Huo,§[a] Junfeng Miao,§[a] Junru Fang,[a] Hu Shi,[a] Juanjuan Wang,[b] and Wei Guo*,[a]

[a] *School of Chemistry and Chemical Engineering, Shanxi University, Taiyuan 030006, China.* [b] *Scientific Instrument Center, Shanxi University, Taiyuan 030006, China*

E-mail: guow@sxu.edu.cn

1. General information and methods

All reagents and solvents were purchased from commercial sources and were of the highest grade. Solvents were dried according to standard procedures. All reactions were magnetically stirred and monitored by thin-layer chromatography (TLC). Flash chromatography (FC) was performed using silica gel 60 (200–300 mesh). Absorption spectra were taken on Varian Carry 4000 spectrophotometer. Fluorescence spectra were taken on Hitachi F-7000 fluorescence spectrometer. The ^{1}H NMR and ^{13}C NMR spectra were taken on a Bruker spectrometer, and recorded at 600 and 150 MHz, respectively. The following abbreviations were used to explain the multiplicities: s = singlet; d = doublet; t = triplet; q = quartet; m = multiplet; br = broad. High resolution mass spectra were obtained on a Varian QFT-ESI mass spectrometer. The imaging assays of cells were performed in Zeiss LSM 880+Airyscan Laser Scanning Confocal Microscope.

2. Synthesis

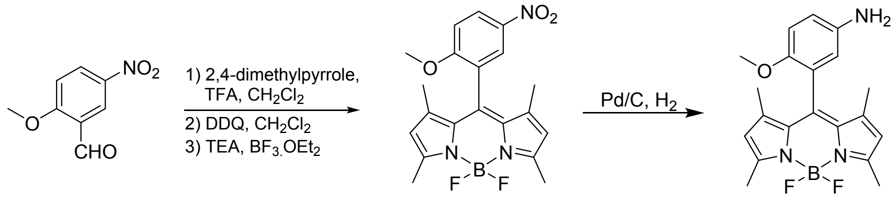

4 3 2

Compound 3: Compound **4** (3.00 g, 16.6 mmol), 2,4-dimethylpyrrole (3.15 g, 33.5 mmol) and CH_2Cl_2 (500 mL) was added to a 1 L reaction flask. The mixture was stirred for 20 min at room temperature under nitrogen. Trifluroacetic acid (30 μL) was added and stirred overnight. After TLC monitoring showed the complete consumption of starting material, a solution of DDQ (2,3-dichloro-5,6-dicyano-1,4-benzoduinone) (3.9 g, 16.6 mmol) in 40 ml of CH_2Cl_2 was added, and stirring was continued for 2 h. The reaction mixture was washed with water, dried over Na_2SO_4, filtered and evaporated. The residue and triethylamine (33 mL) were dissolved in 200 mL of anhydrous CH_2Cl_2, and the solution was stirred at room temperature for 30 min. $BF_3 \cdot OEt_2$ (33 mL) was added, and stirring was continued for 10 min. The reaction

mixture was washed with water and 2 N NaOH. The aqueous solution was extracted with CH_2Cl_2. The combined organic extracts were dried over Na_2SO_4, filtered, and evaporated. The crude compound was purified by column chromatography (EtOAc/PE=1:5) to afford compound **3** as an orange solid (2.3 g, Yield 34.8%). ^{1}H NMR (600 MHz, DMSO) δ 8.47 (dd, J = 9.2, 2.8 Hz, 1H), 8.15 (d, J = 2.8 Hz, 1H), 7.45 (d, J = 9.3 Hz, 1H), 6.20 (s, 2H), 3.94 (s, 3H), 2.46 (s, 6H), 1.42 (s, 6H); ^{13}C NMR (150 MHz, DMSO) δ 161.93, 155.80, 142.50, 142.00, 136.13, 130.97, 127.85, 125.75, 123.74, 122.00, 113.09, 14.73, 14.29.

Compound **2**: A solution of **3** (0.5 g, 1.25 mmol) and 10% Pd-C (0.1 g) in 50 mL CH_2Cl_2 was stirred under H_2 for 3 h at room temperature. The reaction mixture was filtered and the filtrate was evaporated under reduced pressure. The residue was purified by silica gel flash chromatography to give **2** as an orange solid (310 mg, 67% yield). ^{1}H NMR (600 MHz, DMSO) δ 6.91 (d, J = 8.9 Hz, 1H), 6.71 (dd, J = 8.8, 2.8 Hz, 1H), 6.39 (d, J = 2.8 Hz, 1H), 6.14 (s, 2H), 4.84 (s, 2H), 3.62 (s, 3H), 2.43 (s, 6H), 1.52 (s, 6H); ^{13}C NMR (150 MHz, DMSO) δ 157.48, 150.02, 146.93, 145.74, 143.49, 134.20, 126.19, 124.19, 119.25, 117.55, 116.56, 59.18, 17.52, 16.73; ESI-MS: [M+H$^+$], calcd 370.1897, Found 370.1898.

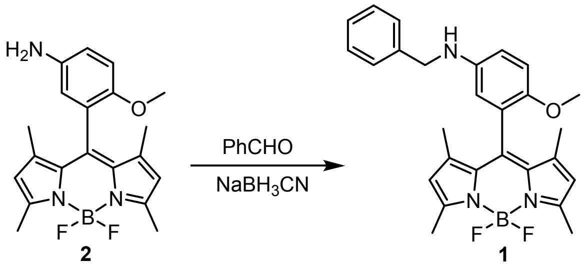

Compound **1**: A solution of **2** (73.8 mg, 0.2 mmol), benzaldehyde (63.7 mg, 0.6 mmol), and AcOH (0.3 mL) in CH_2Cl_2 (8 mL) was stirred at room temperature for 10 min then $NaBH_3CN$ (188.4 mg, 3 mmol) was added, and the obtained solution was stirred for 30 min. The reaction was quenched with water and extracted with $CHCl_3$. The organic layer was washed with brine and dried over Na_2SO_4. Filtration, evaporation and purification of the residue by silica gel chromatography (EtOAc/PE = 1:4) gave **1** as an orange solid (48 mg, 52 % yield). ^{1}H NMR (600 MHz, DMSO) δ 7.31 (d, J = 7.2 Hz, 2H), 7.26 (dd, J = 10.4, 4.9 Hz, 2H), 7.18 (t, J = 7.3 Hz, 1H), 6.94

(d, J = 9.0 Hz, 1H), 6.76 (dd, J = 8.9, 2.8 Hz, 1H), 6.36 (d, J = 2.9 Hz, 1H), 6.10 (s, 2H), 6.07 (t, J = 6.5 Hz, 1H), 4.25 (d, J = 6.4 Hz, 2H), 3.60 (s, 3H), 2.41 (s, 6H), 1.39 (s, 6H); ^{13}C NMR (150 MHz, $CDCl_3$) δ 154.74, 148.69, 142.65, 139.13, 131.42, 128.57, 127.48, 127.27, 124.43, 120.66, 115.17, 114.34, 112.86, 56.08, 49.00, 14.51, 13.76; ^{19}F NMR (376 MHz, $CDCl_3$) δ -145.58, -145.67, -145.76, -145.88, -145.97, -146.06, -146.15, -146.58, -146.67, -146.75, -146.84, -146.96, -147.05, -147.13; ^{11}B NMR (128 MHz, $CDCl^3$) δ 1.06, 0.80, 0.54; ESI-MS: calcd 460.2372, Found 460.2363.

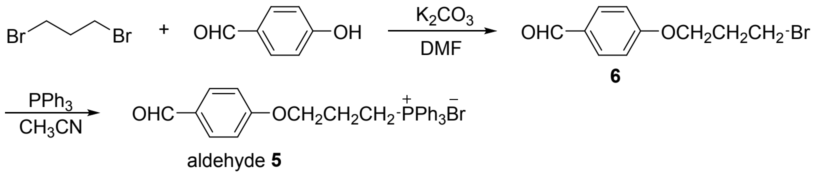

<u>Compound 6:</u> To a solution of 4-hydroxybenzaldehyde (2 g, 16.4 mmol) in 50 mL of DMF were added 1, 3-dibromopropane (3.3 mL, 32.8 mmol) and K_2CO_3 (4.5 g, 32.8 mmol), and the solution was stirred at room temperature for 12 hours. Then the mixture was poured into 150 mL of ice water and extracted with EtOAc for 3 times. The combined organic extracts were dried over Na_2SO_4 and evaporated under reduced pressure to obtain the crude compound, which was purified by column chromatography (EtOAc/PE = 1:3) to afford **6** as a white solid (10.7 g, 92% yield).

<u>Compound 5:</u> Compound **6** (2.6 g, 10 mmol) and triphenylphosphine (7.3 g, 30 mmol) were dissolved in 50 mL of anhydrous acetonitrile, and the solution was heated under reflux for 12 hours. The solvent was evaporated under reduced pressure. The residue was added 5 ml of anhydrous dichloromethane and then poured into 50 ml EtOAc. The white precipitate was collected by filtration. The obtained white solid was purified by silica-gel column chromatography (CH_2Cl_2/MeOH = 30:1) to afford aldehyde **5** as a white powder (4.11, 79% yield). ^{1}H NMR (600 MHz, DMSO-d_6) δ 9.89 (s, 1H), 7.92 – 7.80 (m, 11H), 7.80 – 7.77 (m, 6H), 7.15 – 7.12 (m, 2H), 4.25 (t, J = 5.9 Hz, 2H), 3.81 (t, J = 14.8 Hz, 2H), 2.04 (m, 2H). ^{13}C NMR (150 MHz, DMSO-d_6) δ 194.75, 166.40, 138.38, 137.03, 135.18, 133.66 , 133.22, 121.96, 121.38, 118.40, 70.52, 25.34, 21.18, 20.83.

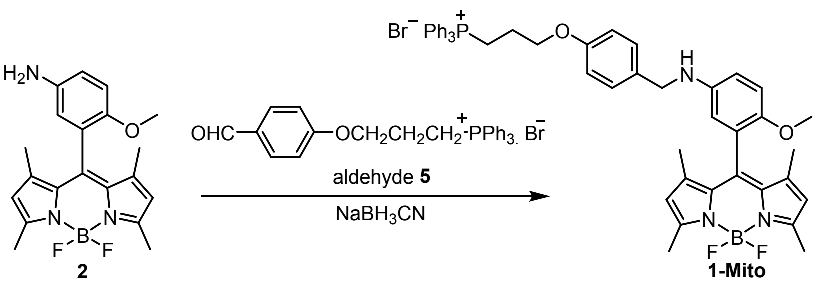

<u>Compound **Mito1**:</u> A solution of **2** (73.8 mg, 0.2 mmol), aldehyde **5** (300 mg, 0.6 mmol), and AcOH (0.3 mL) in solvent (5 mL CH_2Cl_2 and 3 mL MeOH) was stirred at room temperature for 10 min then $NaBH_3CN$ (188.4 mg, 3 mmol) was added, and the obtained solution was stirred for 30 min. The reaction was quenched with water and extracted with $CHCl_3$. The organic layer was washed with brine and dried over Na_2SO_4. Filtration, evaporation and purification of the residue by silica gel chromatography (CH_2Cl_2/EtOH = 40/1) gave **Mito1** as an orange solid (77 mg, 45 % yield). ^{1}H NMR (600 MHz, CD_3OD) δ 7.90 (t, J = 7.3 Hz, 3H), 7.84 (dd, J = 12.7, 7.5 Hz, 6H), 7.76 (td, J = 7.9, 3.5 Hz, 6H), 7.26 (d, J = 8.4 Hz, 2H), 6.95 (d, J = 8.9 Hz, 1H), 6.86 – 6.81 (m, 3H), 6.45 (d, J = 2.7 Hz, 1H), 5.99 (s, 2H), 4.25 (s, 2H), 4.11 (t, J = 5.6 Hz, 2H), 3.69 (s, 3H), 3.63 – 3.57 (m, 2H), 2.46 (s, 6H), 2.15 (d, J = 6.2 Hz, 2H), 1.47 (s, 6H); ^{13}C NMR (150 MHz, CD_3OD) δ 150.80, 155.92, 149.46, 145.29, 144.46, 141.58, 136.51, 136.49, 134.98, 134.91, 133.99, 131.73, 131.65, 129.91, 125.30, 121.67, 120.16, 119.58, 117.00, 115.71, 115.46, 114.39, 67.87, 56.66, 23.93, 20.31, 19.96, 14.62, 14.15; ^{19}F NMR (376 MHz, $CDCl_3$) δ -145.60, -145.69, -145.78, -145.90, -145.99, -146.08, -146.17, -146.57, -146.66, -146.74, -146.83, -146.95, -147.04, -147.13; ^{11}B NMR (128 MHz, $CDCl_3$) δ 1.05, 0.79, 0.53; ESI-MS: calcd 778.3540, Found 778.3560.

3. Preparation of the test solution

Stock solution of **1** or **Mito1** in CH_3CN (2 mM) was used to prepare the working solutions in PBS (50 mM, pH 7.4, containing 20% CH_3CN) with a final concentration of 4.0 μM. For assays in chemical system, the NO stock solution in deionized water was used, which was prepared by bubbling NO gas into a NaOH solution to eliminate NO_2 generated from the reaction of NO and O_2, and then into deoxygenated deionized

water for 30 min. The concentration of the resulting NO stock solution was determined to be 1.8 mM by Griess method. For assays in cells, a commercially available NO donor NOC-9 (dissolved in 0.1 M NaOH solution) was used. For assays in chemical system, a $ONOO^-$ solution, which was synthesized according to a reported procedure,[1] was used, and its concentration was determined using an extinction coefficient of 1670 $M^{-1}cm^{-1}$ at 302 nm. For cell imaging assays, $ONOO^-$ was generated from a commercially available $ONOO^-$ donor SIN-1 (dissolved in 0.1 M NaOH solution). $O_2^{\bullet-}$ was prepared by adding KO_2 (7.1 mg) and 18-Crown-6 (1 equiv) to dry dimethyl sulfoxide (5 mL) and stirring vigorously for 10 min. $HO^{\bullet}$ was generated *in situ* by the Fenton reaction, and its concentration was equal to the Fe(II) concentration. $^1O_2$ was generated *in situ* by adding NaClO solution into H_2O_2 solution (10 eq), and its concentration was equal to the NaClO concentration. H_2O_2 solution was prepared by dilution of commercial H_2O_2 solution in deionized water, and its concentration was determined by using an extinction coefficient of 43.6 $M^{-1}cm^{-1}$ at 240 nm. NaClO solution was prepared by the dilution of commercial NaClO solution in deionized water, and its concentration was determined using an extinction coefficient of 350 $M^{-1}cm^{-1}$ at 292 nm. The aqueous solutions of $NaNO_2$ was freshly prepared and used as NO_2^- source. The aqueous solutions of K^+, Ca^{2+}, Na^+, Mg^{2+}, Al^{3+}, Zn^{2+}, Fe^{2+}, Fe^{3+}, Cu^+, and Cu^{2+} were freshly prepared from their chloride salts. The aqueous solutions of Cys/GSH and the DMSO solutions of DHA/AA/MGO were freshly prepared. For spectra studies, various analytes, except $^{\bullet}OH$ and $^1O_2$, were directly added to the solution of **1** or **Mito1** (4 μM) in PBS (50 mM, pH 7.4, containing 20% CH_3CN), and then fluorescence spectra were recorded in the indicated time points. For $^{\bullet}OH$ or $^1O_2$, **1** or **Mito1** and H_2O_2 were premixed, and then Fe^{2+} or ClO^- was added to the mixture.

4. Cell culture and fluorescence imaging

4.1 Cell culture

The HeLa cell line, Raw 264.7 macrophage cell line, THP-1 cell line, and SKOV-3

cell line were kindly provided by Key Laboratory of Chemical Biology and Molecular Engineering of Ministry of Education (China). HeLa cells, Raw 264.7 macrophages and SKOV-3 cells were grown in Dulbecco's modification of Eagle's medium (DMEM) supplemented with 10 % FBS (Fetal Bovine Serum) at 37 ºC in humidified environment of 5% CO_2. THP-1 cell were cultivated in ATCC-formulated Roswell Park Memorial Institute medium (RPMI-1640) with 0.05 mM 2-mercaptoethanol and 10% FBS at 37 ºC in humidified environment of 5% CO_2. Cells were plated on glass bottom cell culture dish (30 mm) and allowed to adhere for 12 hours. Before experiments, cells were washed with phosphate buffer saline (PBS) 3 times.

4.2 Imaging exogenous and endogenous NO in HeLa cells and RAW264.7 macrophage cells

To test the selectivity of **1** or **Mito1** for NO in cell environment, HeLa cells were pretreated with **1** or **Mito1** (2 μM) in PBS for 20 min, and then treated with NOC-9 (25 μM) (commercially available NO donor), SIN-1 (10 μM) (commercially available $ONOO^-$ donor), and representative ROS, including H_2O_2 (50 μM), ClO^- (50 μM), respectively, for 20 min. For imaging of intracellular basal NO, Raw 264.7 cells were directly treated with **1** or **Mito1** (2 μM, 20 min) in PBS; for inhibition assays, Raw 264.7 cells were pretreated with AG (0.5 mM, 6 h) in PBS, and then treated with **1** or **Mito1** (2 μM, 20 min). For imaging of stimulator-induced NO, Raw 264.7 cells were pretreated with stimulators LPS (20 μg/mL)/INF-γ (150 units/mL) for 6 h in PBS and then treated with **1** or **Mito1** (2 μM, 20 min); for inhibition assays, Raw 264.7 cells were pretreated in PBS with LPS (20 μg/mL)/INF-γ (150 units/mL) for 6 h in the presence of AG (0.5 mM), and then treated with **1** or **Mito1** (2 μM, 20 min). After each treatment, the cells were washed with PBS 3 times. Emission was collected at 493−600 nm (λ_{ex}: 488 nm).

4.3 Cell costaining studies.

To evaluate the subcellular localization of **1** or **Mito1**, HeLa cells were incubated with **1** or **Mito1** (both 2.0 μM) and MitoTracker™ Deep Red FM (0.3 μM) (or LysoTracker® Deep Red (0.07 μM) in PBS for 30 min, and after washing with PBS 3

times, the cells were treated with NOC-9 (25 μM, 20 min) to light up the fluorescence. To test whether **1** is localized in both mitochondria and lysosomes, HeLa cells were co-incubated with **1** (2.0 μM), MitoTracker™ Deep Red FM (0.3 μM), and LysoTracker® Deep Red (0.07 μM) in PBS for 30 min, and after washing with PBS 3 times, the cells were treated with NOC-9 (30 μM, 20 min) to light up the fluorescence. For **1** and **Mito1**, emission was collected at 493–600 nm (λ_{ex} = 488 nm). For MitoTracker™ Deep Red FM and LysoTracker® Deep Red, emission was collected at 638-747 nm (λ_{ex} = 633 nm).

4.4 Polarization of THP-1 into M1 and M2 macrophages and repolarization of M2 macrophages to M1 macrophages

Human THP-1 monocytes are differentiated into macrophages by 24 h incubation with 150 nM phorbol 12-myristate 13-acetate (PMA) followed by 24 h incubation in RPMI 1640 medium. Macrophages thus obtained were polarized in M1 macrophages by incubation with 20 ng/mL of IFN-γ and 10 pg/mL of LPS in RPMI 1640 medium for 24 h. Macrophage M2 polarization was obtained by incubation with 20 ng/mL of interleukin 4 (IL-4) and 20 ng/mL of interleukin 13 (IL-13) in RPMI 1640 medium for 72 h. Repolarization of macrophages from M2 phenotype to M1 phenotype were achieved by treating M2 macrophages with 50 ng/mLof IFN-γ for 24 h and then with 200 ng/mL of LPS for 48 h in RPMI 1640 medium. After each treatment, the cells were washed thoroughly with PBS to remove all stimulus.

4.5 Discriminating M1 and M2 macrophages in term of their difference in NO level and imaging NO communication during the phagocytosis of SKOV-3 cancer cells by macrophages in a co-culture system

For imaging NO in M1 and M2 macrophages, these cells were directly treated with **1** in PBS for 20 min, and for inhibition assay, M1 macrophages were first treated with AG (0.5 mM) in PBS for 12 h, and then treated with **1** for 20 min. For imaging NO in repolarized M1 macrophages from M2 macrophages, M2 macrophages were treated with IFN-γ (50 ng/mL, 24 h) and LPS (200 ng/mL, 48 h) successively in RPMI 1640 medium, followed by the treatment with **1** (2 μM, 20 min) in PBS. For imaging NO

communication between M1 macrophages and SKOV-3 cells, M1 macrophages were first stained with commercial blue-fluorescent nucleus dye DAPI (1 μg/mL, 20 min) in RPMI 1640 medium, and then co-cultured with **1**-loaded SKOV-3 cells (2 μM, 20 min) in RPMI 1640 medium for 12 h. For inhibition assay, M1 macrophages were pretreated with AG (0.5 mM, 6 h), and after stained with DAPI (1 μg/mL, 20 min), the cells were co-cultured with **1**-loaded SKOV-3 cells (2 μM, 20 min) in RPMI 1640 medium for 12 h. For imaging NO communication between M2 macrophages and SKOV-3 cells, M2 macrophages were first stained with DAPI (1 μg/mL, 20 min), and then co-cultured with **1**-loaded SKOV-3 cells (2 μM, 20 min) in RPMI 1640 medium for 12 h. For imaging NO communication between the repolarized M1 macrophages and SKOV-3 cells, M2 macrophages were first repolarized to M1 macrophages by IFN-γ/LPS treatment described above, and then co-cultured with **1**-loaded SKOV-3 cells (2 μM, 20 min) in RPMI 1640 medium for 12 h. Note that the use of DAPI dye in the assays allowed us to distinguish between M1 or M2 macrophages and SKOV-3 cells in the co-culture system. After each treatment, the cells should be washed thoroughly with PBS to remove all stimulus. For probe channel, emission was collected at 493−600 nm (λ_{ex} = 488 nm); for DAPI channel, emission was collected at 410−485 nm (λ_{ex} = 405 nm).

5. MTT assays

HeLa Cells were seeded in 96-well microplates in DMEM medium supplemented with 10 % FBS (Fetal Bovine Serum) at 37 ºC in humidified environment of 5% CO_2. After 24 h of cell attachment, the plates were washed with PBS, followed by addition of increasing concentrations of **1** (2−50 μM) or **Mito1** (1−14 μM) in DMEM. The cells were then incubated at 37 ºC in an atmosphere of 5% CO_2 and 95% air for 24 h, followed by standard MTT assays (n= 6). Untreated assays (n = 6) were also conducted under the same conditions.

6. Supplementary Spectra

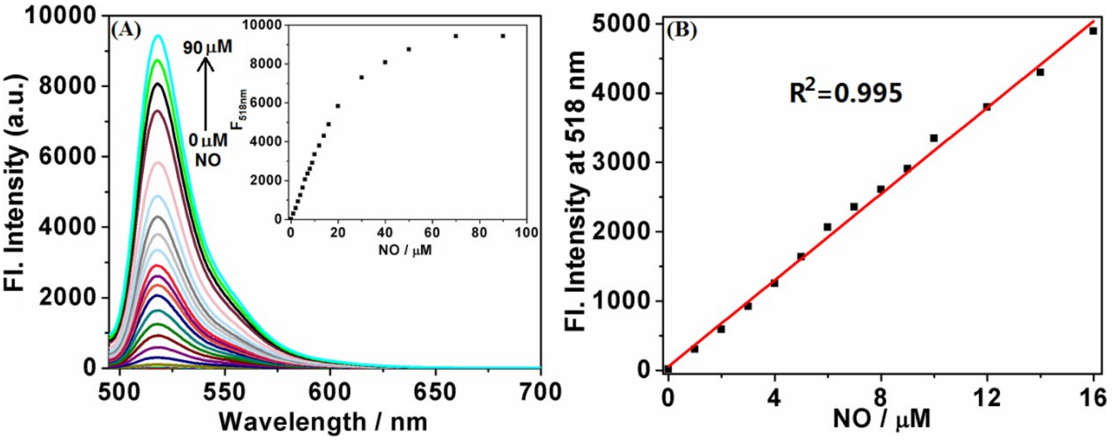

Figure S1 (A) Fluorescence spectra changes of **1** (4 μM) treated with the increased concentrations of NO solution (0–90 μM) in aerobic condition. (B) Plot of the fluorescence intensities of **1** (4 μM) at 518 nm as a function of NO concentrations (0–16 μM). Conditions: PBS (50 mM, pH 7.4, containing 20% CH_3CN); λ_{ex} =485 nm; λ_{em} = 518 nm; Slits: 5/10 nm; voltage: 600 V. Note that the detection limit (DL) for NO was calculated to be 0.8 nM based on $3\sigma/k$, and for N_2O_3 being 0.4 nM in term of the equation of $4NO + O_2 = 2N_2O_3$.

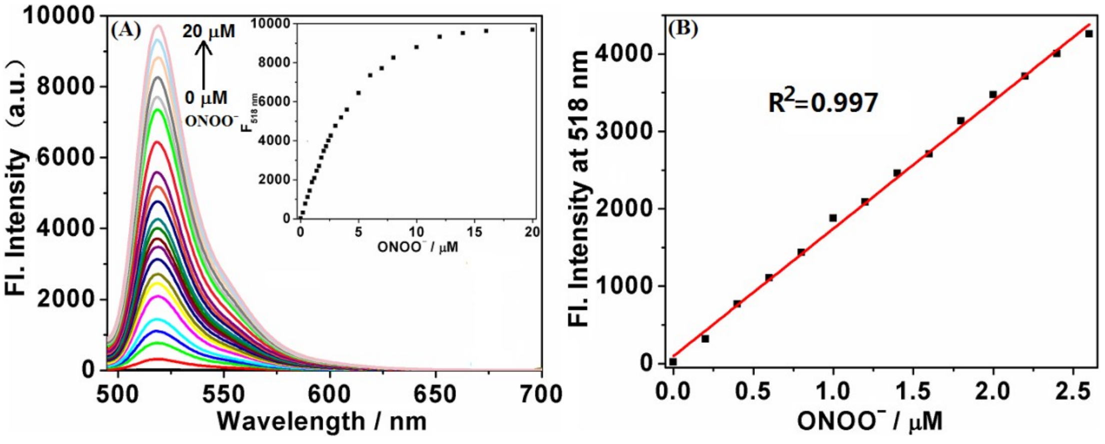

Figure S2 (A) Fluorescence spectra changes of **1** (4 μM) treated with the increased concentrations of $ONOO^-$ (0–20 μM). (B) Plot of the fluorescence intensities of **1** (4 μM) at 518 nm as a function of $ONOO^-$ concentrations (0–2.6 μM). Conditions: PBS (50 mM, pH 7.4, containing 20% CH_3CN); λ_{ex} = 485 nm; λ_{em} = 518 nm; Slits: 5/10 nm; voltage: 600 V.

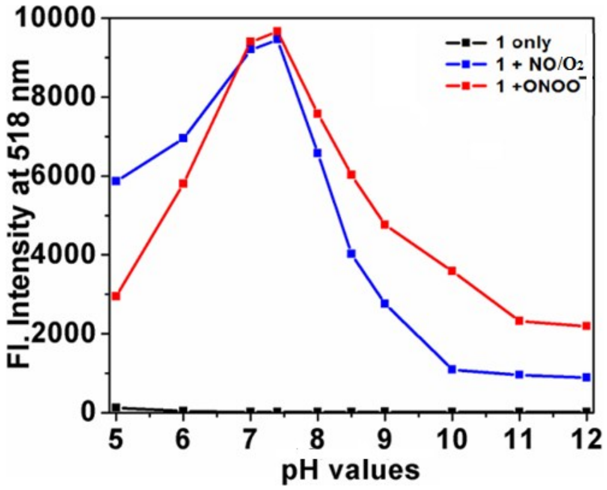

Figure S3 The emission intensities at 518 nm for **1** (4 μM) in the absence and presence of NO (50 μM) or $ONOO^-$ (20 μM) at varied pH values in aerobic condition. Condition: B-R buffer (20 mM, pH = 5-12, containing 20% CH_3CN). λ_{ex} = 485 nm; λ_{em} = 518 nm; Slits: 5/10 nm; voltage: 600 V.

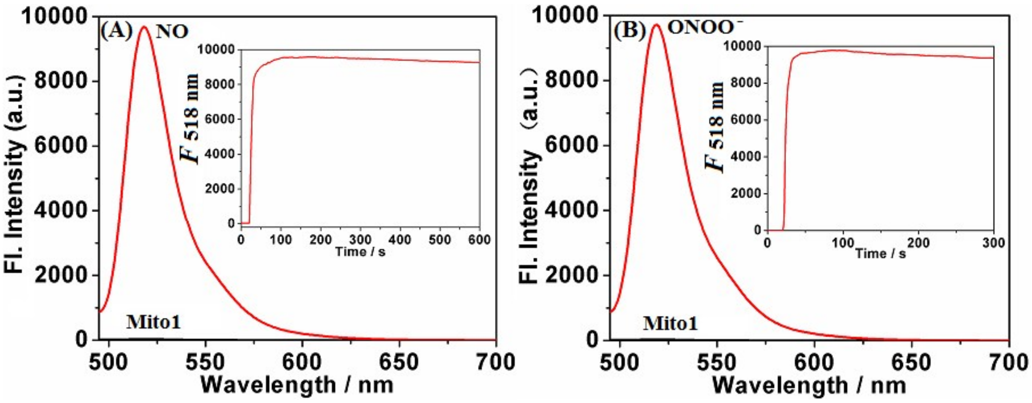

Figure S4 Fluorescence spectra of **Mito1** (4 μM) in the absence and presence of 50 μM of NO (A) or 20 μM of $ONOO^-$ (B) in aerobic condition. Inset: the corresponding time-dependent fluorescence intensity changes. Conditions: PBS (50 mM, pH 7.4, containing 20% CH_3CN); λ_{ex} =485 nm; λ_{em} = 518 nm; Slits: 5/10 nm; voltage: 600 V.

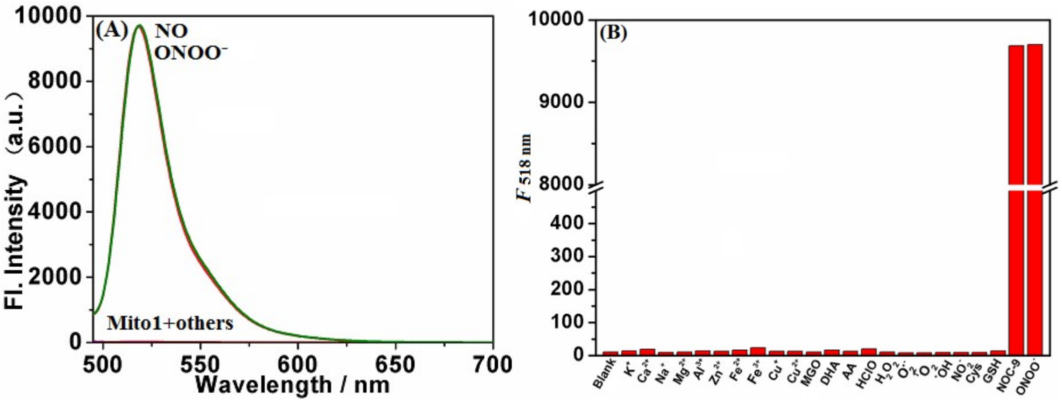

Figure S5 Fluorescence spectra (A) and intensities (B) of **Mito1** (4 μM) treated with various biologically relevant species in aerobic condition, including 1 mM of K^+, Ca^{2+}, Na^+, Mg^{2+}, Al^{3+}, Zn^{2+}, Fe^{2+}, Fe^{3+}, Cu^+, Cu^{2+}, MGO, DHA, and AA; 100 μM of HClO, H_2O_2, $O_2^{\bullet-}$, $^1O_2$, $HO^\bullet$, and NO_2^-; 1 mM of Cys and GSH; 50 μM of NO and 20 μM of $ONOO^-$, at the time point of 2 min. Conditions: PBS (50 mM, pH 7.4, containing 20% CH_3CN); λ_{ex} = 485 nm; λ_{em} = 518 nm; Slits: 5/10 nm; voltage: 600 V.

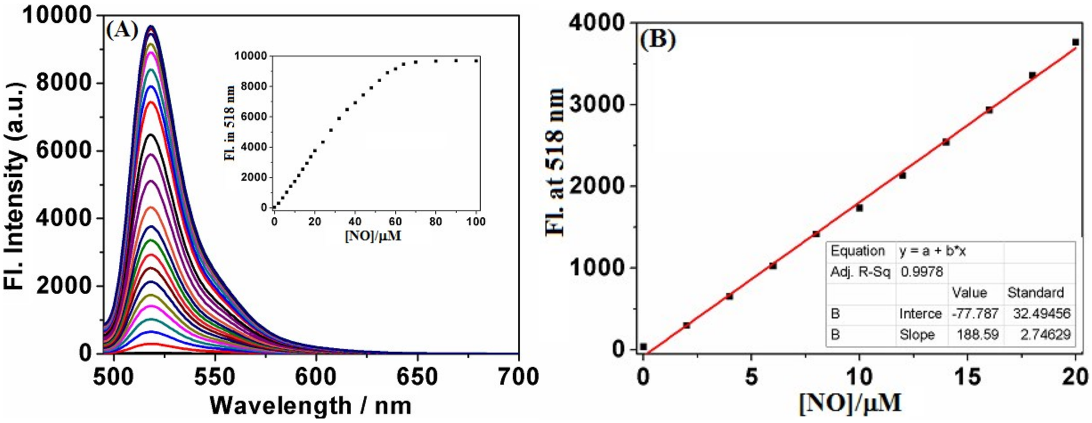

Figure S6 (A) Fluorescence spectra changes of **Mito1** (4 μM) treated with the increased concentrations of NO solution (0–100 μM) in aerobic condition. (B) Plot of the fluorescence intensities of **Mito1** (4 μM) at 518 nm as a function of NO concentrations (0–20 μM). The detection limit of **Mito1** for NO was calculated to be 3.8 nM based on $3\sigma/k$, and for N_2O_3 being 1.9 nM in term of the equation of $4NO + O_2 = 2N_2O_3$. Conditions: PBS (50 mM, pH 7.4, containing 20% CH_3CN); λ_{ex} =485 nm; λ_{em} = 518 nm; Slits: 5/10 nm; voltage: 600 V.

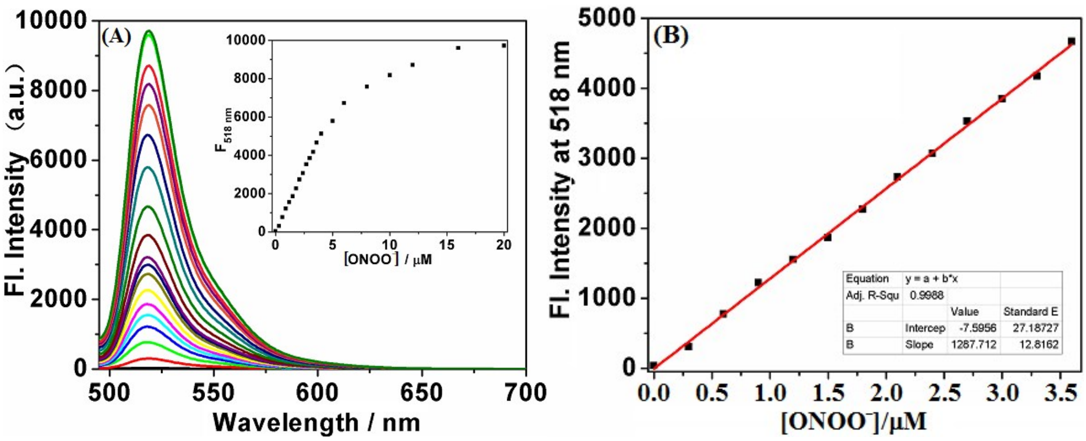

Figure S7 (A) Fluorescence spectra changes of **Mito1** (4 μM) treated with the increased concentrations of $ONOO^-$ (0–20 μM). (B) Plot of the fluorescence intensities of **Mito1** (4 μM) at 518 nm as a function of $ONOO^-$ concentrations (0–3.6 μM). The detection limit of **Mito1** for $ONOO^-$ was calculated to be 0.98 nM based on $3\sigma/k$. Conditions: PBS (50 mM, pH 7.4, containing 20% CH_3CN); λ_{ex} =485 nm; λ_{em} = 518 nm; Slits: 5/10 nm; voltage: 600 V.

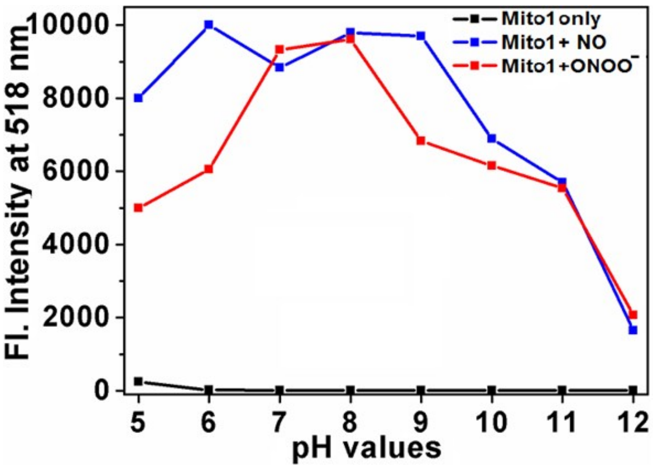

Figure S8 The emission intensities at 518 nm for **Mito1** (4 μM) in the absence and presence of NO (50 μM) and $ONOO^-$ (20 μM) at varied pH values in aerobic condition. Condition: B-R buffer (20 mM, pH = 5-12, containing 20% CH_3CN). λ_{ex} = 485 nm; λ_{em} = 518 nm; Slits: 5/10 nm; voltage: 600 V.

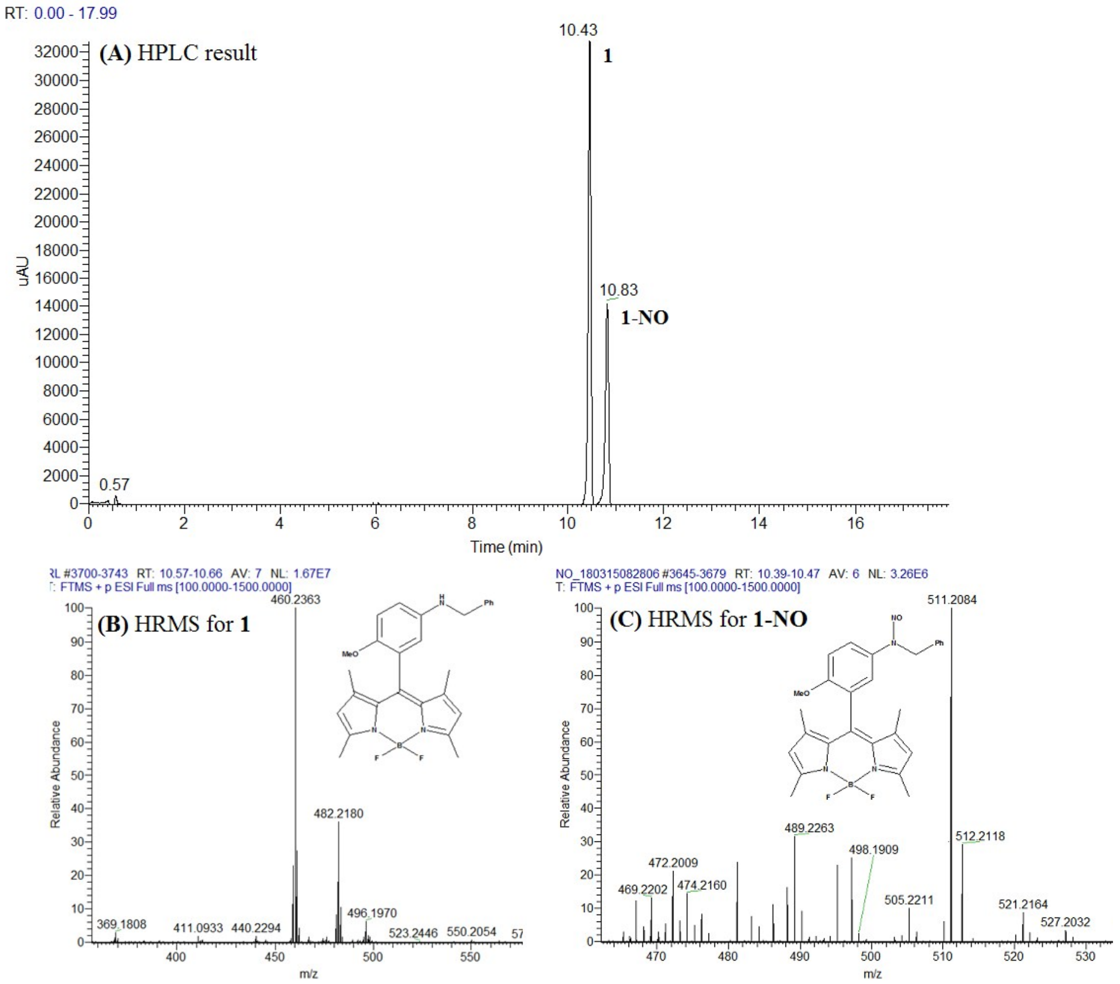

Figure S9 HPLC-MS results of **1** treated with excess of NO solution in aerobic condition in PBS (50 mM, pH 7.4, containing 20% CH_3CN).

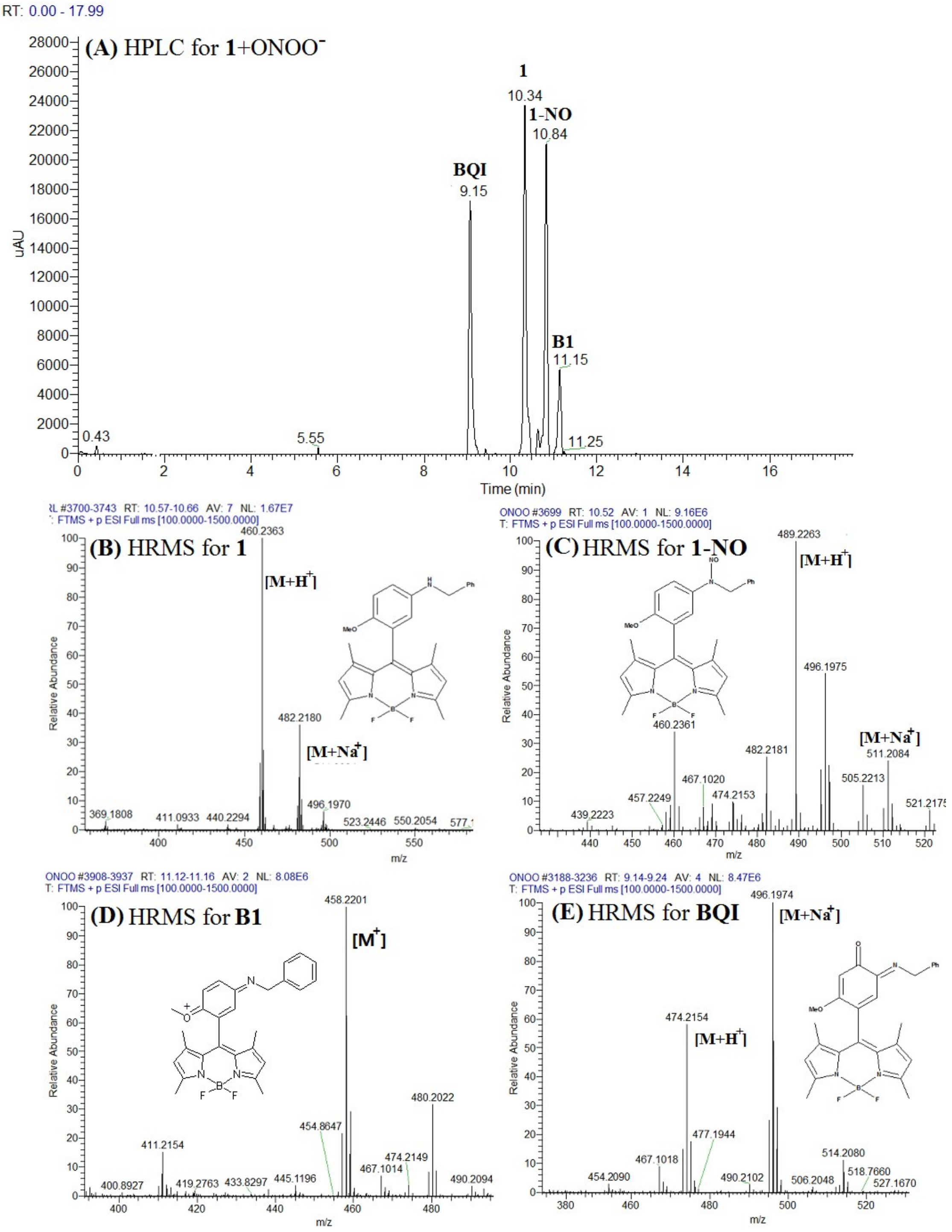

Figure S10 HPLC-MS results of **1** treated with excess of ONOO⁻ in PBS (50 mM, pH 7.4, containing 20% CH_3CN).

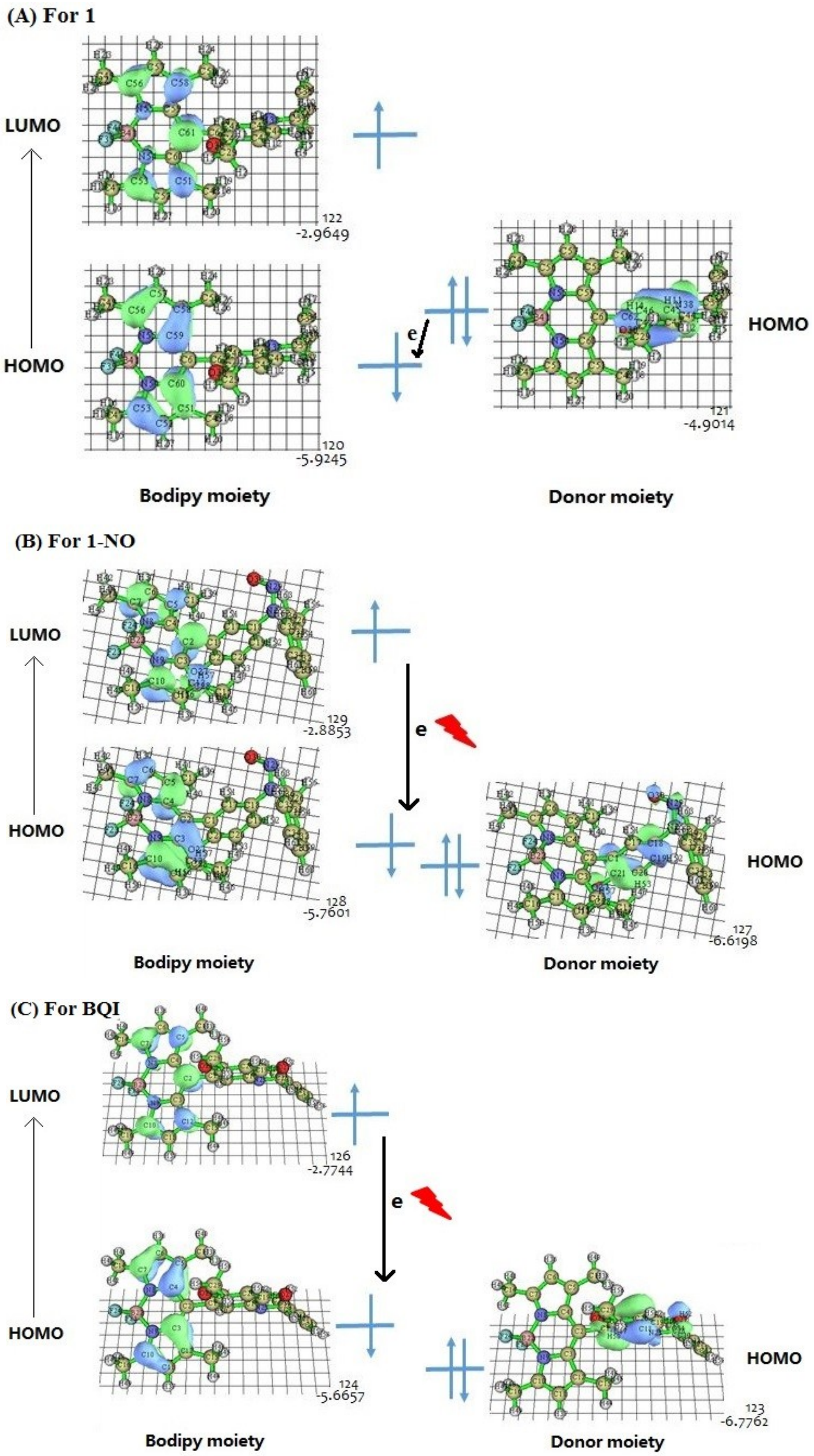

Figure S11 Frontier orbital energy representation of the PeT process in **1** (A), **1-NO** (B), and **BQI** (C). All the theoretical studies were performed in PCM model in water by Gaussian 09 suite[2] with Becke's three-parameter hybrid exchange function with

Lee-Yang-Parr gradient-corrected correlation functional (B3LYP functional) and 6-31+G* basis set.[3] All the local minima structures were confirmed by the absence of an imaginary mode in vibrational analysis calculations.

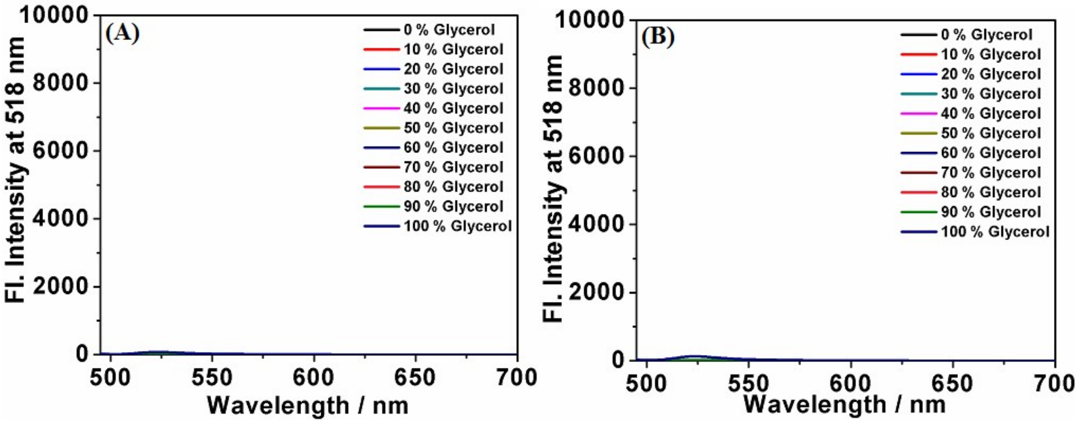

Figure S12 The fluorescence spectra of **1** (A) and **Mito1** (B) (both 4μM) in the deionized water-glycerol systems (0% – 100% of glycerol) with varied viscosity. Condition: λ_{ex} = 485 nm; λ_{em} = 518 nm; Slits: 5/10 nm; voltage: 600 V. The results indicate that the fluorescence of **1** or **Mito1** is indeed quenched by PeT process, rather than rotation or vibration-relevant nonradiative process.

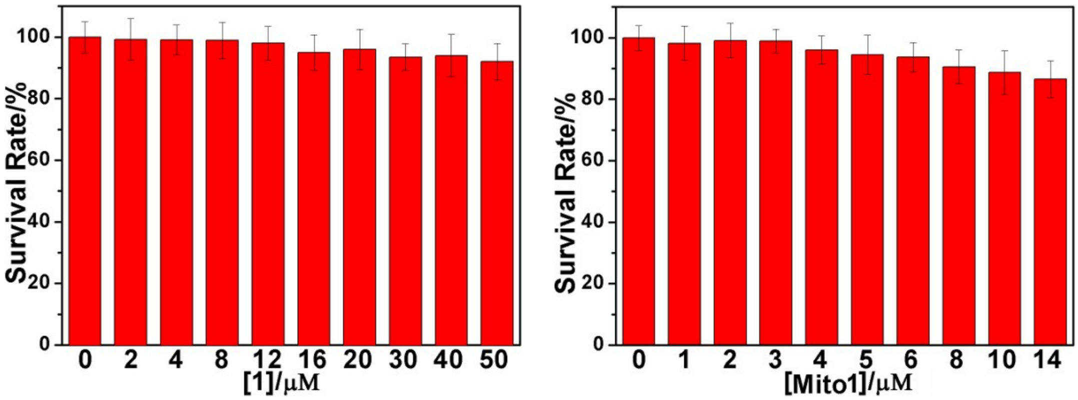

Figure S13 Percentage of viable HeLa cells after treated with increasing concentrations of **1** and **Mito1** for 24 hours.

7. ^{1}H NMR, ^{13}C NMR and HRMS Charts

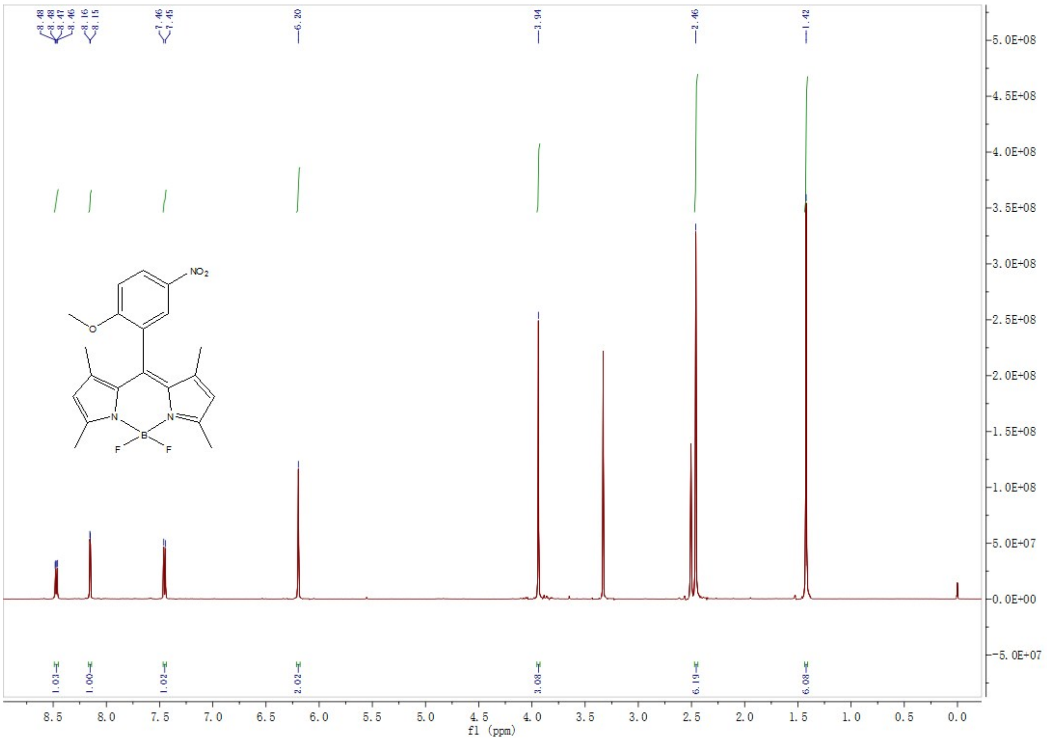

Figure S14 ^{1}H NMR chart of **3** (DMSO-d_6, 600 MHz).

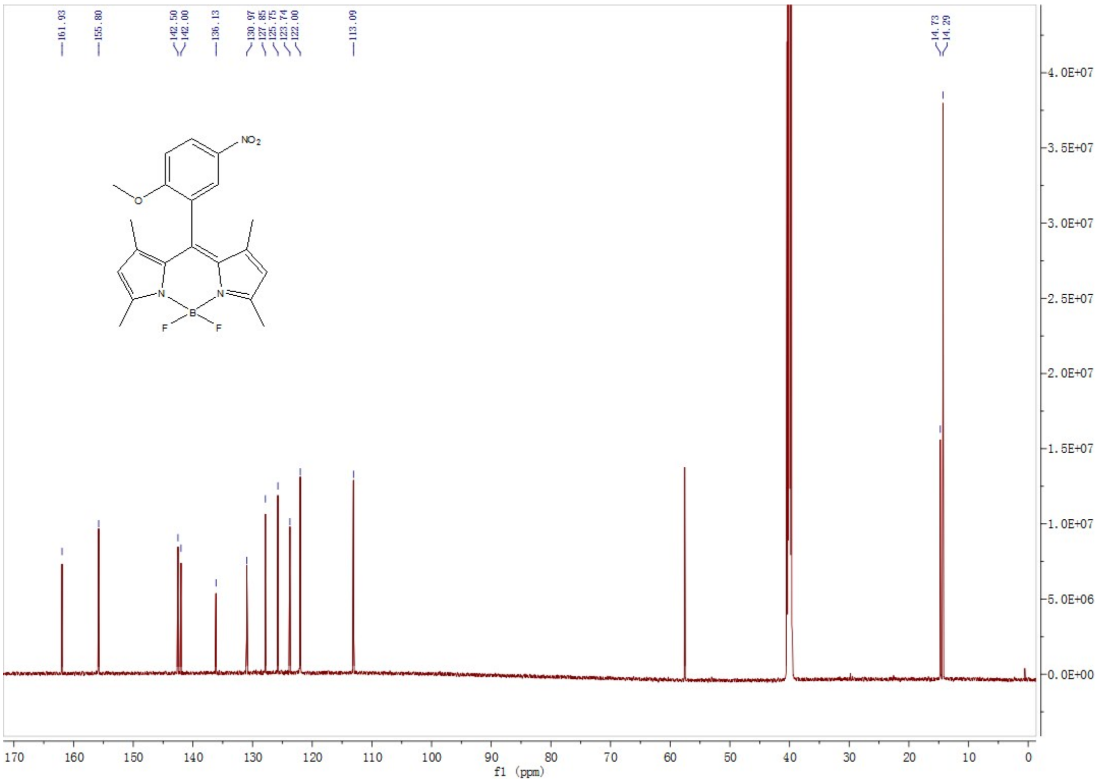

Figure S15 ^{13}C NMR chart of **3** (DMSO-d_6, 150 MHz).

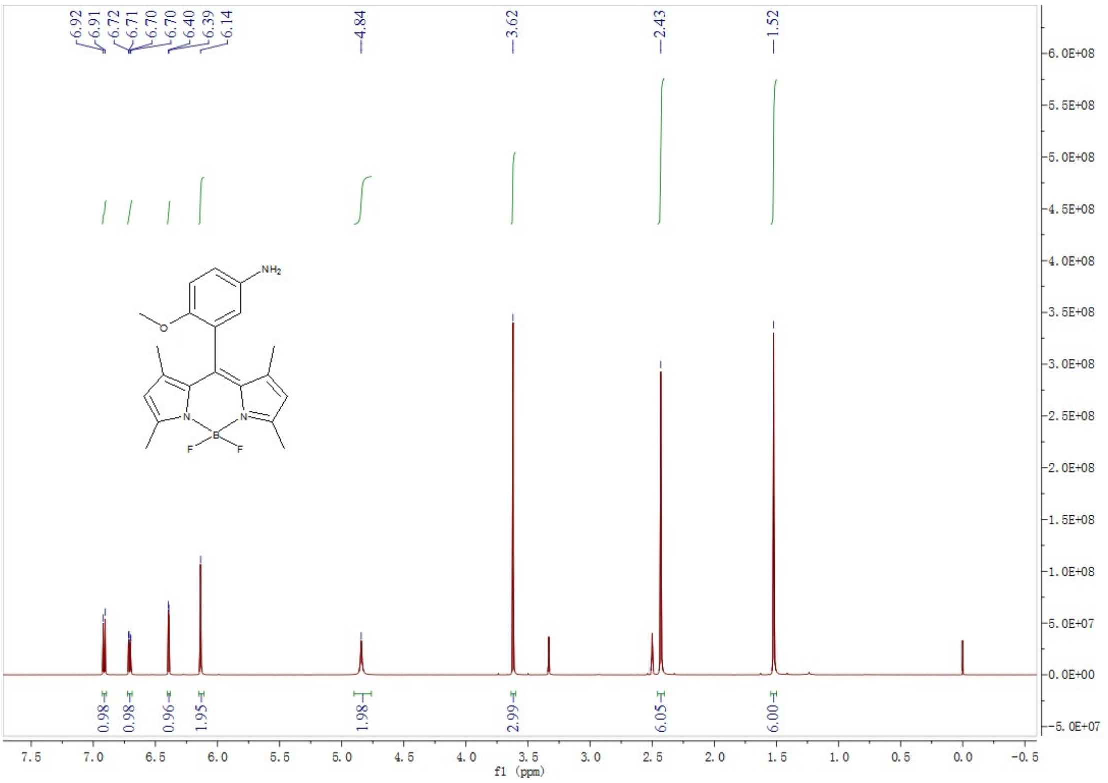

Figure S16 ^{1}H NMR chart of **2** (DMSO-d_6, 600 MHz).

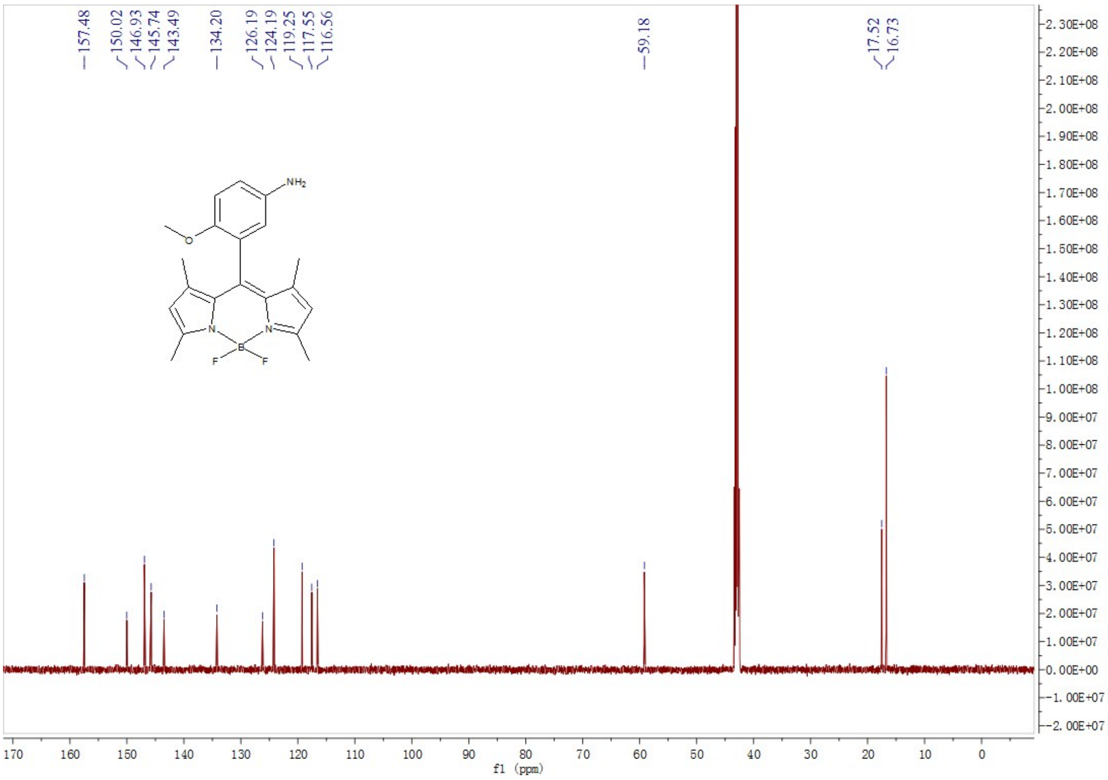

Figure S17 ^{13}C NMR chart of **2** (DMSO-d_6, 150 MHz).

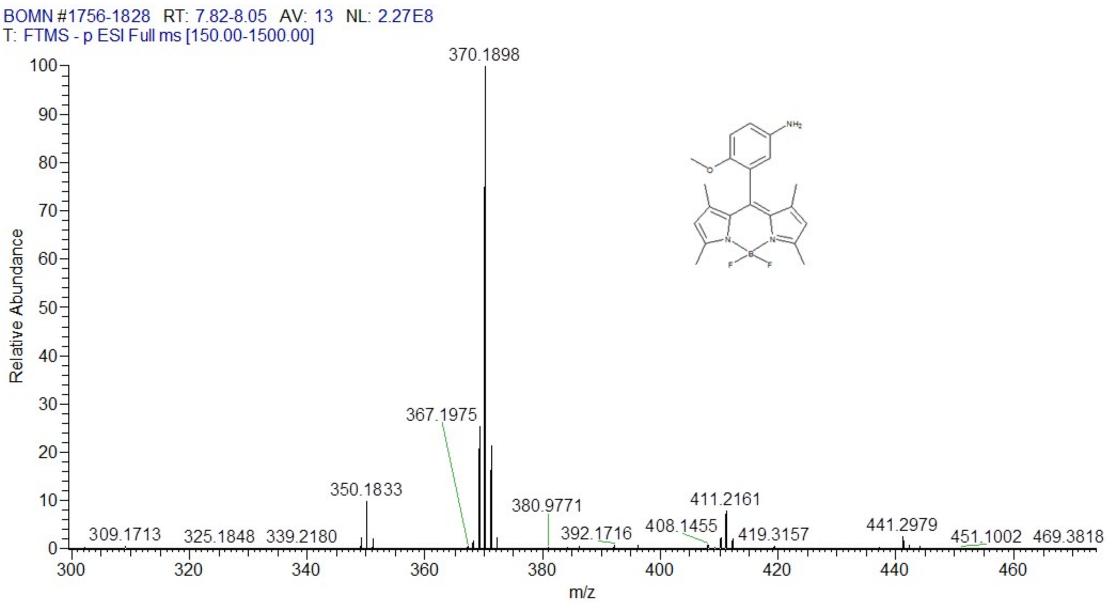

Figure S18 HRMS chart of **2**.

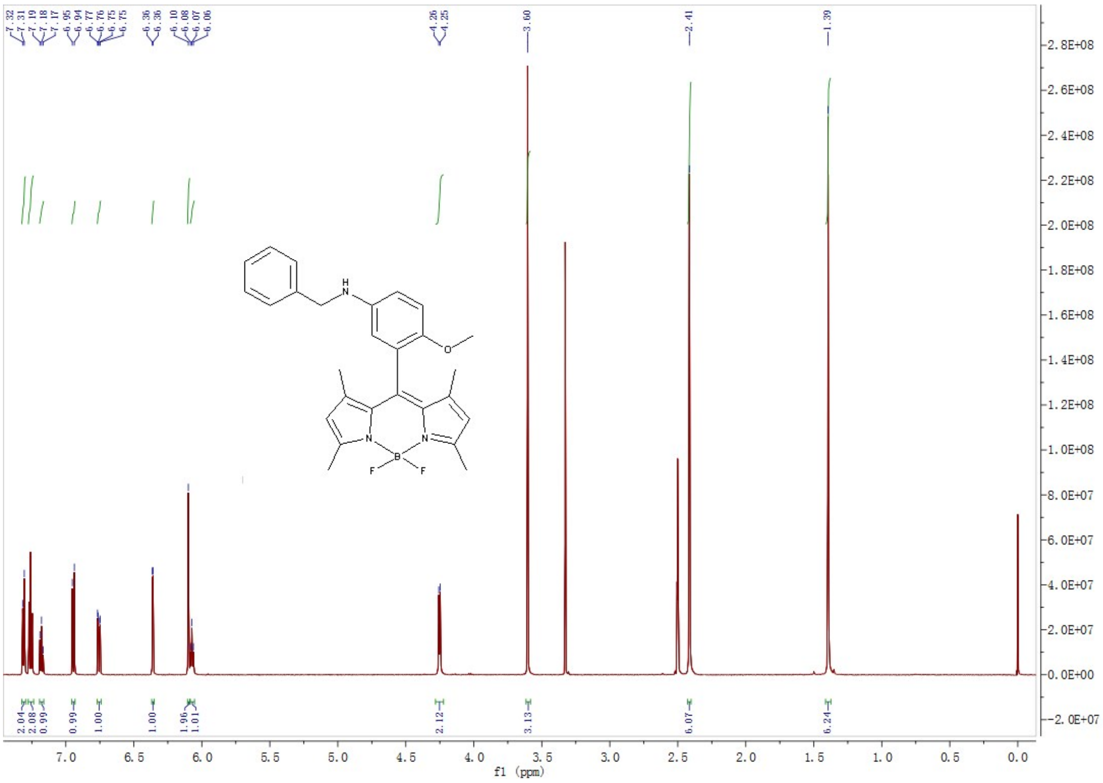

Figure S19 ^{1}H NMR chart of **1** (DMSO-d_6, 600 MHz).

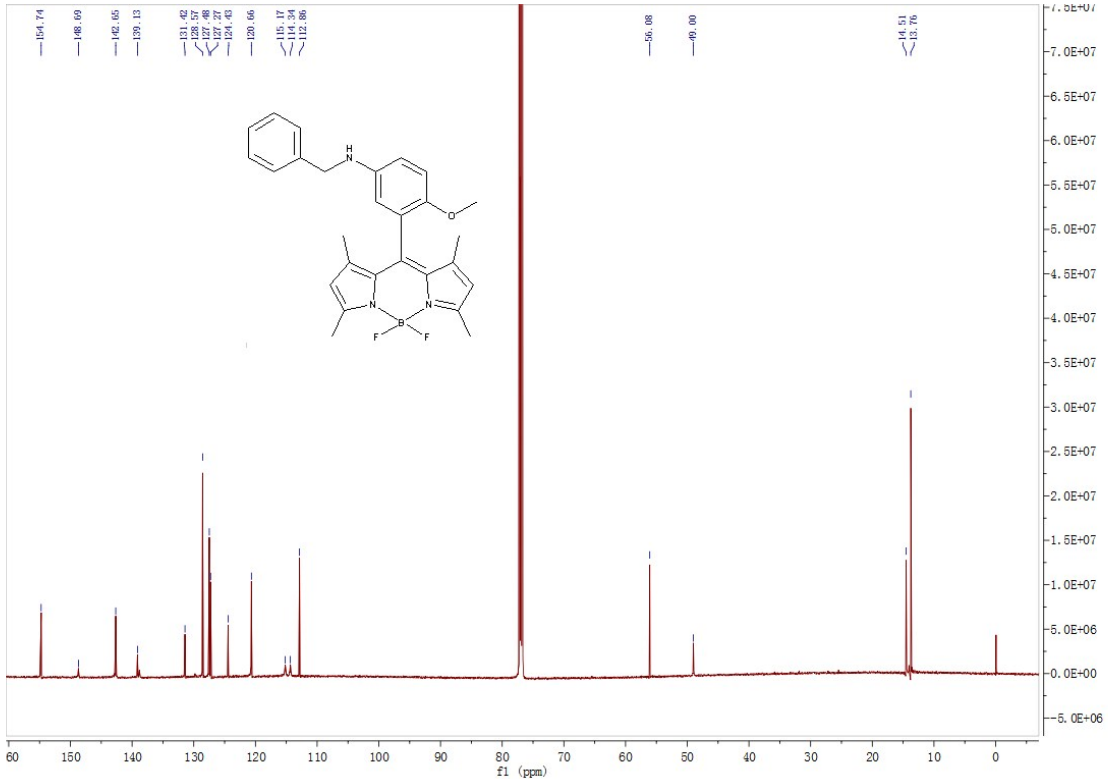

Figure S20 ^{13}C NMR chart of **1** ($CDCl_3$, 150 MHz).

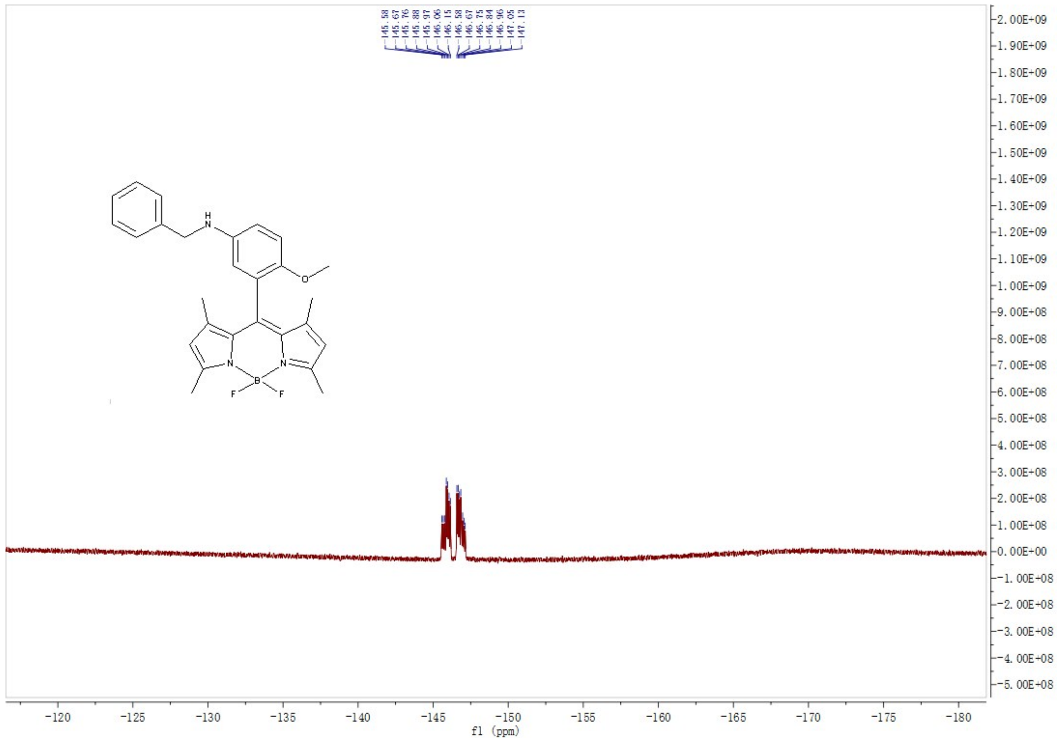

Figure S21 ^{19}F NMR chart of **1** (376 MHz, $CDCl_3$).

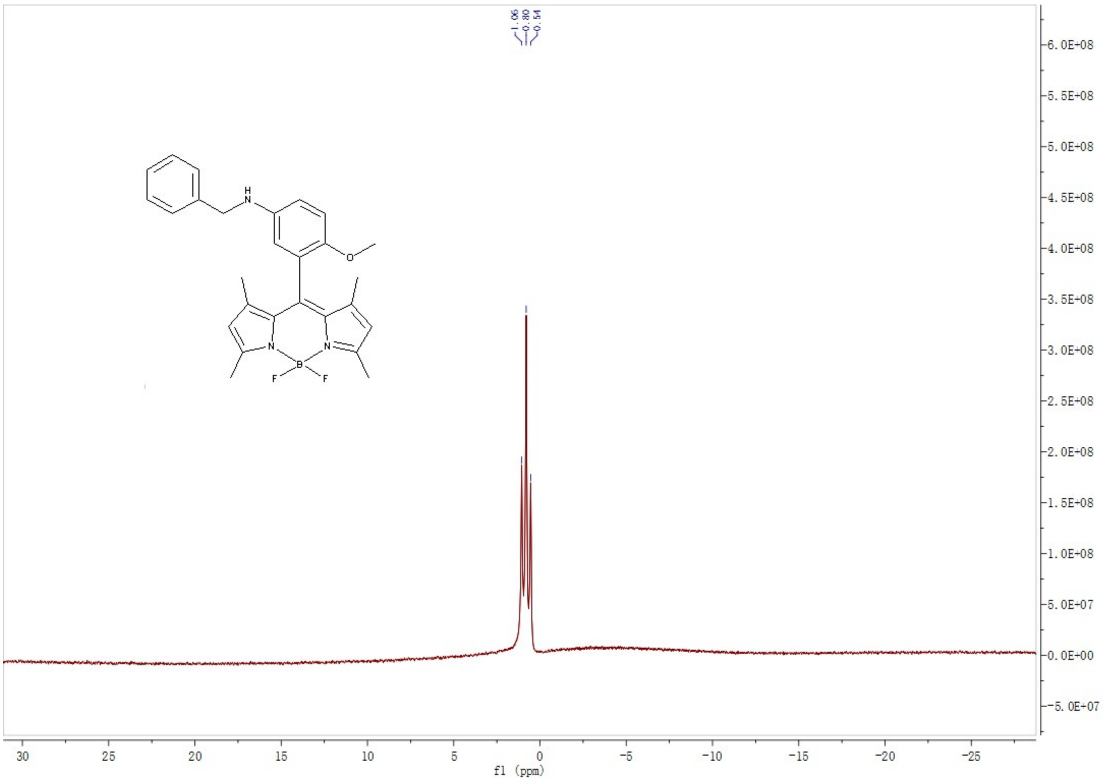

Figure S22 ^{11}B NMR chart of **1** (128 MHz, $CDCl_3$).

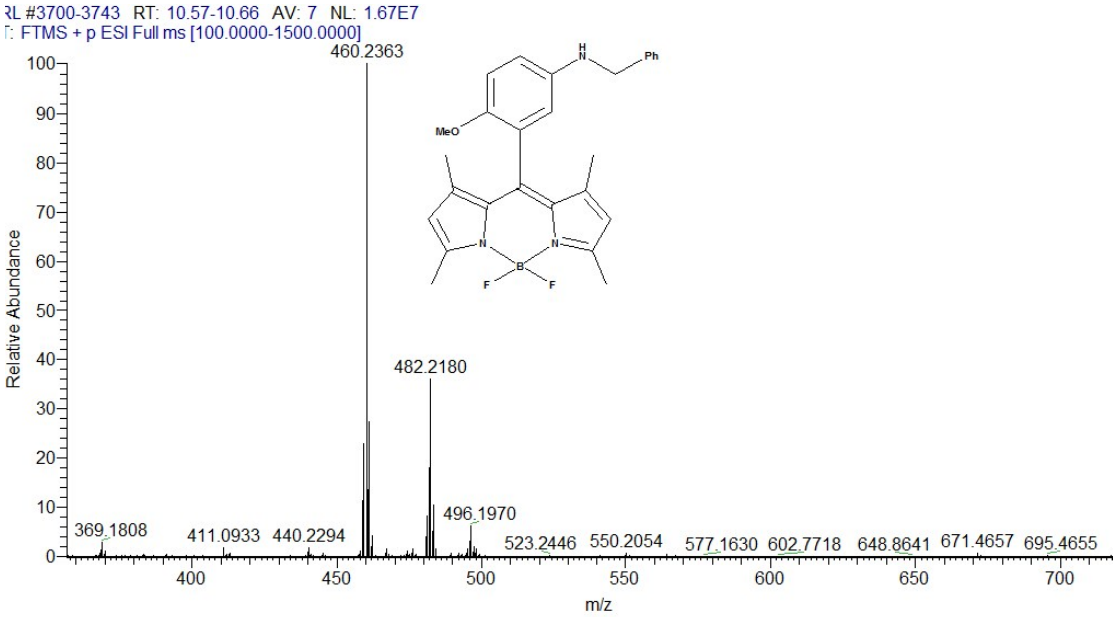

Figure S23 HRMS chart of **1**.

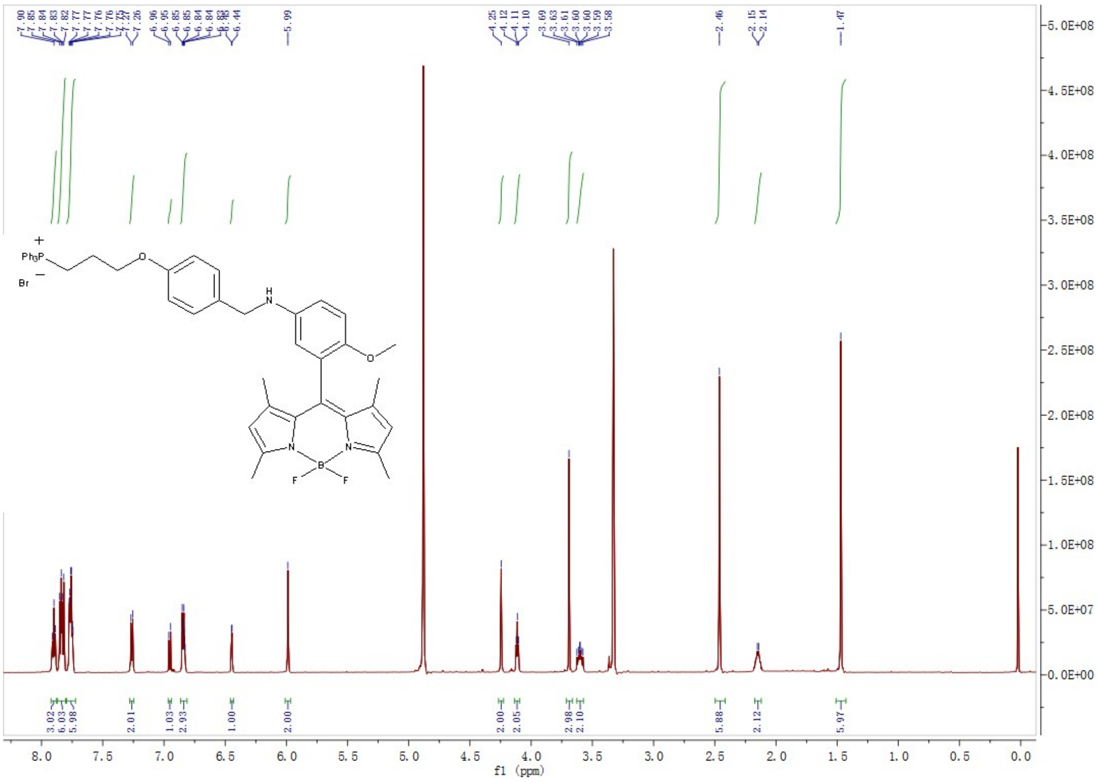

Figure S24 ^{1}H NMR chart of **Mito1** (CD_3OD, 600 MHz).

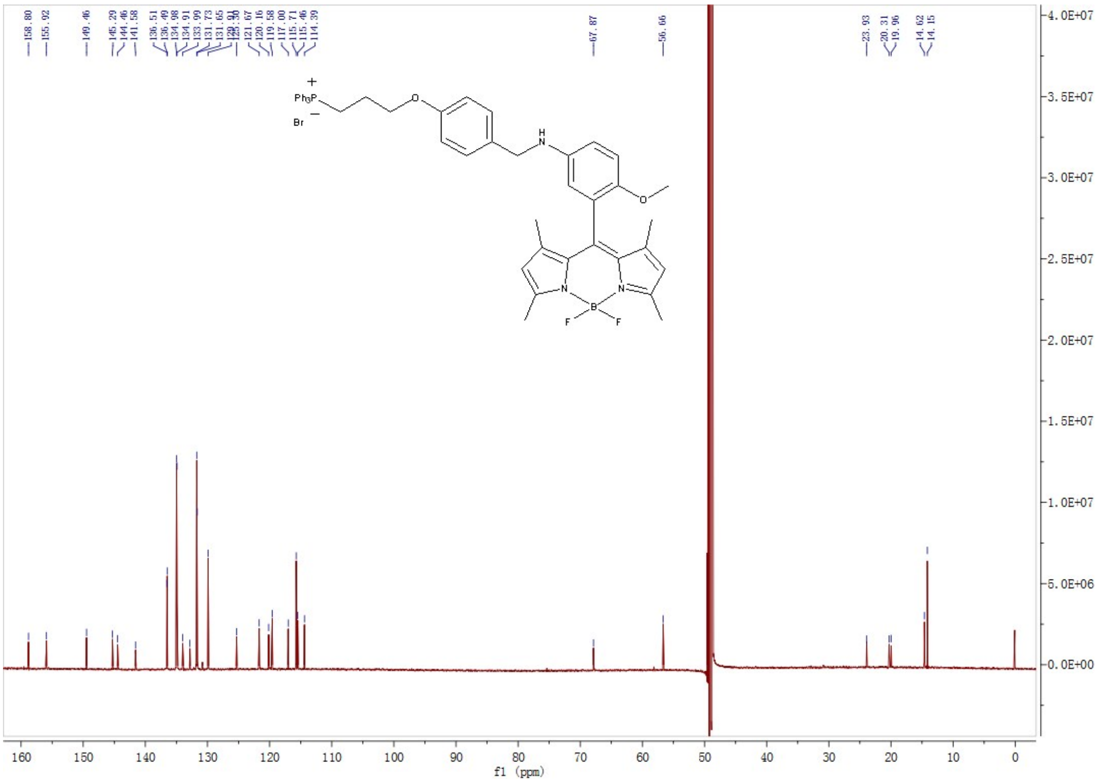

Figure S25 ^{13}C NMR chart of **Mito1** (CD_3OD, 150 MHz).

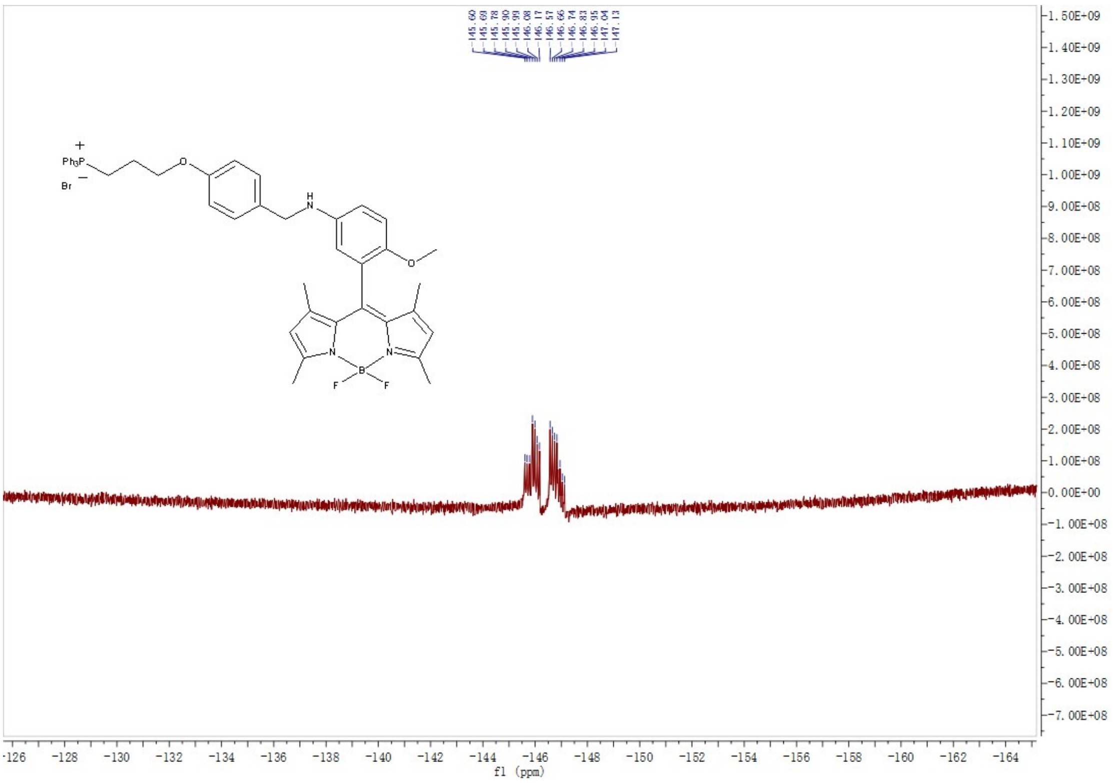

Figure S26 ^{19}F NMR chart of **Mito1** (376 MHz, $CDCl_3$).

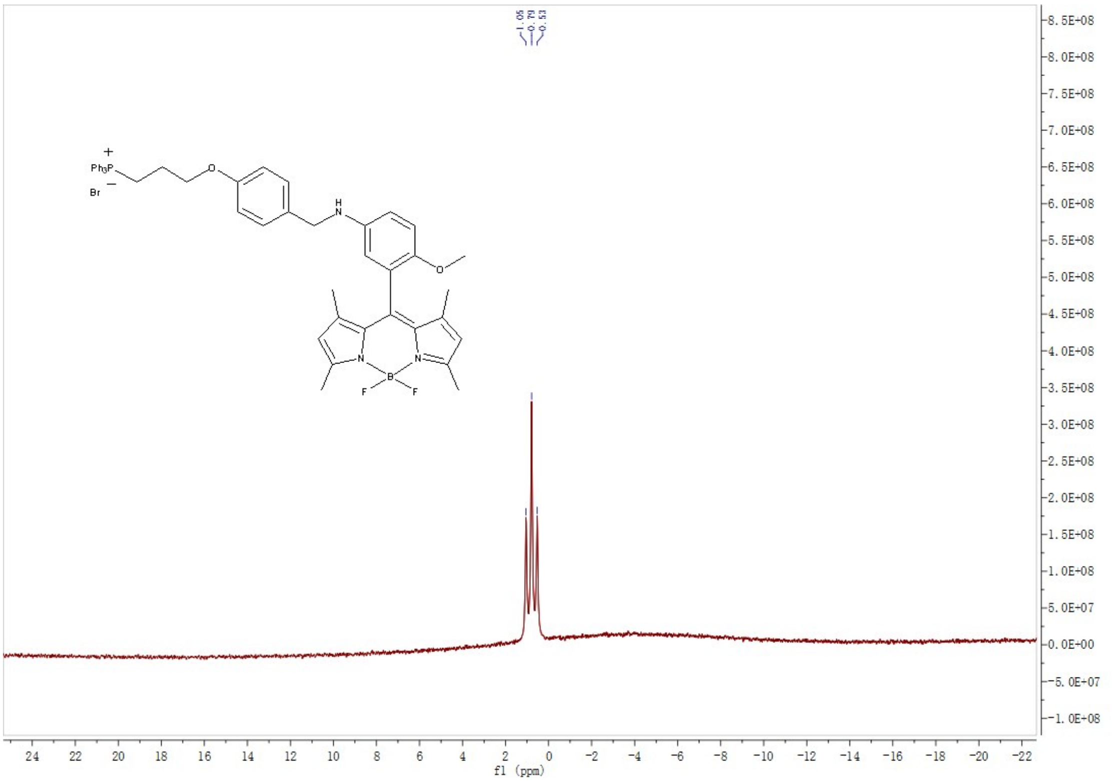

Figure S27 ^{11}B NMR chart of **Mito1** (128 MHz, $CDCl_3$).

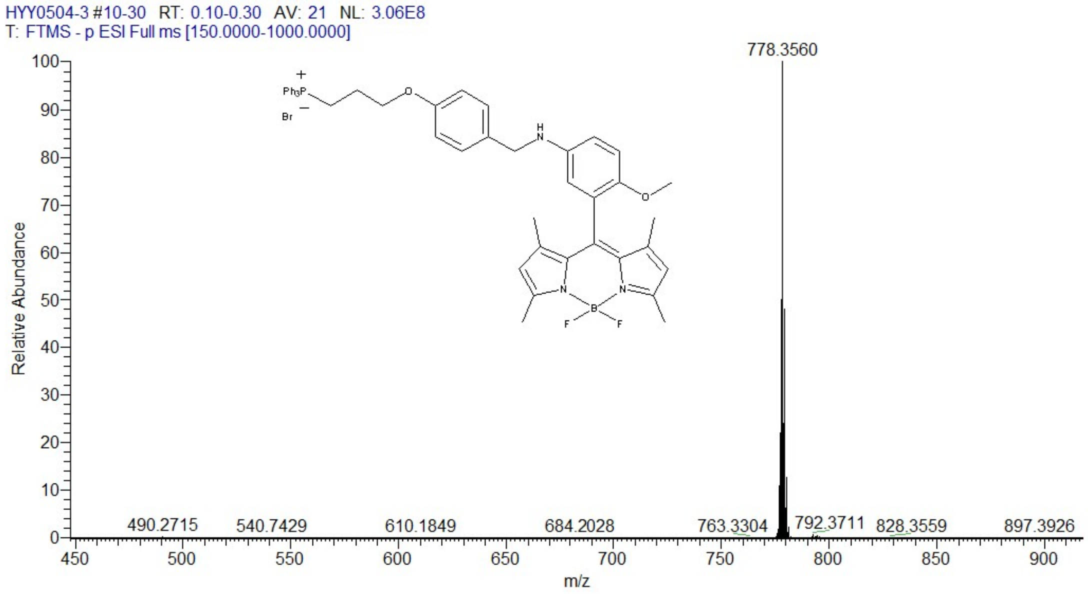

Figure S28 HRMS chart of **Mito1**.

8. References

1. R. M Uppu, W. A. Pryor, *Anal. Biochem.*, 1996, **236**, 242.

2. M. J. Frisch, et al. Gaussian 09, Revision D.01, Gaussian Inc., Wallingford, CT (2009).

3. V. V. Pavlishchuk and A. W. Addison, *Inorg. Chim. Acta*, 2000, **298**, 97.
